# A Geometrically-Constrained Mathematical Model of Mammary Gland Ductal Elongation Reveals Novel Cellular Dynamics within the Terminal End Bud

**DOI:** 10.1371/journal.pcbi.1004839

**Published:** 2016-04-26

**Authors:** Ingrid Paine, Arnaud Chauviere, John Landua, Amulya Sreekumar, Vittorio Cristini, Jeffrey Rosen, Michael T. Lewis

**Affiliations:** 1 Lester and Sue Smith Breast Center, Baylor College of Medicine, Houston, Texas, United States of America; 2 Department of Molecular and Cellular Biology, Baylor College of Medicine, Houston, Texas, United States of America; 3 University Grenoble Alpes, TIMC-IMAG (DyCTiM), Grenoble, France; 4 Department of NanoMedicine and Biomedical Engineering, University of Texas Medical School at Houston, Houston, Texas, United States of America; 5 Brown Foundation Institute of Molecular Medicine, University of Texas Medical School at Houston, Houston, Texas, United States of America; 6 Department of Imaging Physics, University of Texas MD Anderson Cancer Center, Houston, Texas, United States of America; 7 Department of Mathematics, Faculty of Science, King Abdulaziz University, Jeddah, Saudi Arabia; 8 Department of Molecular and Cellular Biology and Radiology, Baylor College of Medicine, Houston, Texas, United States of America; Rutgers University, UNITED STATES

## Abstract

Mathematics is often used to model biological systems. In mammary gland development, mathematical modeling has been limited to acinar and branching morphogenesis and breast cancer, without reference to normal duct formation. We present a model of ductal elongation that exploits the geometrically-constrained shape of the terminal end bud (TEB), the growing tip of the duct, and incorporates morphometrics, region-specific proliferation and apoptosis rates. Iterative model refinement and behavior analysis, compared with biological data, indicated that the traditional metric of nipple to the ductal front distance, or percent fat pad filled to evaluate ductal elongation rate can be misleading, as it disregards branching events that can reduce its magnitude. Further, model driven investigations of the fates of specific TEB cell types confirmed migration of cap cells into the body cell layer, but showed their subsequent preferential elimination by apoptosis, thus minimizing their contribution to the luminal lineage and the mature duct.

## Author Summary

Our paper describes a mathematical model of mammary ductal elongation during pubertal development. We make several conclusions that will be of interest to scientists studying mammary gland biology, epithelial tube formation, and branching morphogenesis. First, our model indicates that a common measurement of developmental outgrowth (‘percent fat pad filled’) underestimates the total growth and leads to mischaracterization of mutant phenotypes. Second, we show that cap cells, a population enriched with putative mammary stem cells, do not contribute to the luminal lineage as previously hypothesized. Further, we find that a high percentage of proliferation in these cells is not used productively to actually form the mammary duct. We believe our model has future application to other branching organs and also for the modeling of disease states in the breast.

## Introduction

Mathematical models have been used to inform basic biological research for well over 100 years [1]. Today, mathematical and computational models are particularly useful as a complementary approach to lab-based studies in developmental biology. Such models have been developed to investigate morphogenetic phenomena [2, 3] in a broad sense, and in particular, for developmental pattern formation (e.g. [4, 5]), vascular branching (e.g. [6]), cell morphology (e.g. [7]), angiogenesis (e.g. [8, 9]), as well as both normal (e.g. [10–13]) and cancerous (e.g. [14–16]) development and regeneration/renewal (e.g. [17–19]) in many tissue types, including breast [20, 21]. Branching morphogenesis specifically has been modeled (see, e.g. [22] for a review) in the lung [23], kidney [24], vasculature structures (e.g. [6]), plants (e.g. [25, 26]), and to a certain extent, the mammary gland [11, 27–30]). In most of these studies, the modeling framework includes reaction-diffusion equations to account for soluble factors driving the growth (e.g. VGEF, FGF) or playing a regulatory role (e.g. Wnt, TGFβ).

With respect to the normal mammary gland, several groups have modeled normal physiological processes. Grant et al. developed a model of mammary ductal morphogenesis adapted from a cellular-automaton model of vascular morphogenesis [27]. While this model was capable of approximating mammary branching gross morphology, it did not take into account the arrangement of the cells at small scales, or use any experimentally derived measurements. Nelson et al. also investigated branching processes in the mammary gland with engineered epithelial tubes. Although the process described does not directly mimic mammary development in vivo, they were able to mathematically model spacing of separate ductal units [31]. At smaller scales of interest, Tang et al. created an agent based model of breast acinus formation in vitro, in which they were able to determine the proliferation and apoptotic dynamics required for proper lumen formation and DCIS development [11], whereas Rejniak et al. have proposed a sophisticated single deformable cell based model to derive conditions for acinus structure and lumen stability [30].

Although the works cited above have been successful in modeling particular aspects of normal mammary development (i.e. ductal tree and acinus formation and some of the corresponding regulation), there are currently no published models of mammary ductal elongation, a critical process required for, and intimately coupled with, branching morphogenesis [32]. In this work, we sought to fill this gap by generating a mathematical model of mammary ductal elongation that not only incorporates the structure responsible for the majority of pubertal development, the terminal end bud (TEB) [33], but also accounts for all of the main biological processes occurring within the TEB that are required for its growth.

In the mammary gland, the rudimentary mammary duct formed during embryonic development remains relatively quiescent until puberty. Ductal elongation during puberty is driven by, and dependent upon, TEBs [32]. TEBs are bulbous, multi-layered structures that direct the growth of the duct throughout the fat pad during puberty. Mammary gland organogenesis in the virgin animal is dependent largely on this process of ductal elongation. During elongation TEBs are responsible for fat pad invasion, bifurcation of main ducts, lumen formation, basement membrane deposition, and the recruitment of periductal stroma and a blood supply. These critical functions, and the geometrically-constrained manner in which they are accomplished, make the TEB an ideal structure on which to base a mathematical model for ductal elongation. In addition, the ductal elongation rates in the murine mammary gland far exceed the allometric growth of other organs in the postnatal mouse, making this structure of unique interest. Lastly, TEBs have been postulated as a major site susceptible to carcinogenesis, leading to tumors later in life [34, 35]. These characteristics make the TEB not only a good model for normal ductal morphogenesis, but also an ideal ground state for modeling breast tumorigenesis.

In order to begin to model this structure we base the equations on the following established TEB biology. TEBs have two main cellular compartments. The outer monolayer of the TEB is known as the cap cell layer, which differentiates into the myoepithelial cell layer of the mature duct [33, 36, 37], and is a putative reservoir for mammary stem cells [33, 38, 39]. Underlying the cap cell layer is a multi-cellular layer called the body cell layer. The cells in the body cell layer give rise to the layer of luminal epithelial cells lining the interior of the duct [36, 37]. Apoptosis in the body cell layer has been postulated as the primary mechanism of lumen formation. Finally, high levels of proliferation in these two compartments of the TEB produce the cells that form the mature duct.

We employed an investigative and integrative approach [19] using repeated iterations of biological measurements and corresponding parameter estimation to identify key processes required for ductal elongation. Through mathematical output calculation, we show that evaluation of just five experimentally measurable parameters, (cell type-specific proliferation, cell type-specific apoptosis, cell size and number, and duct tortuosity), are sufficient to predict ductal displacement and elongation rates accurately. These data, and the associated modeling iterations required to interpret them, indicate that current common interpretations of developmental phenotypes can be inaccurate and require careful reconsideration. This model serves as a baseline model upon which agent-based, cellular automaton, and other mathematical models can be built to explore both normal mammary gland and breast cancer development.

## Results

### Derivation of Base Equations

**Fig 1:**
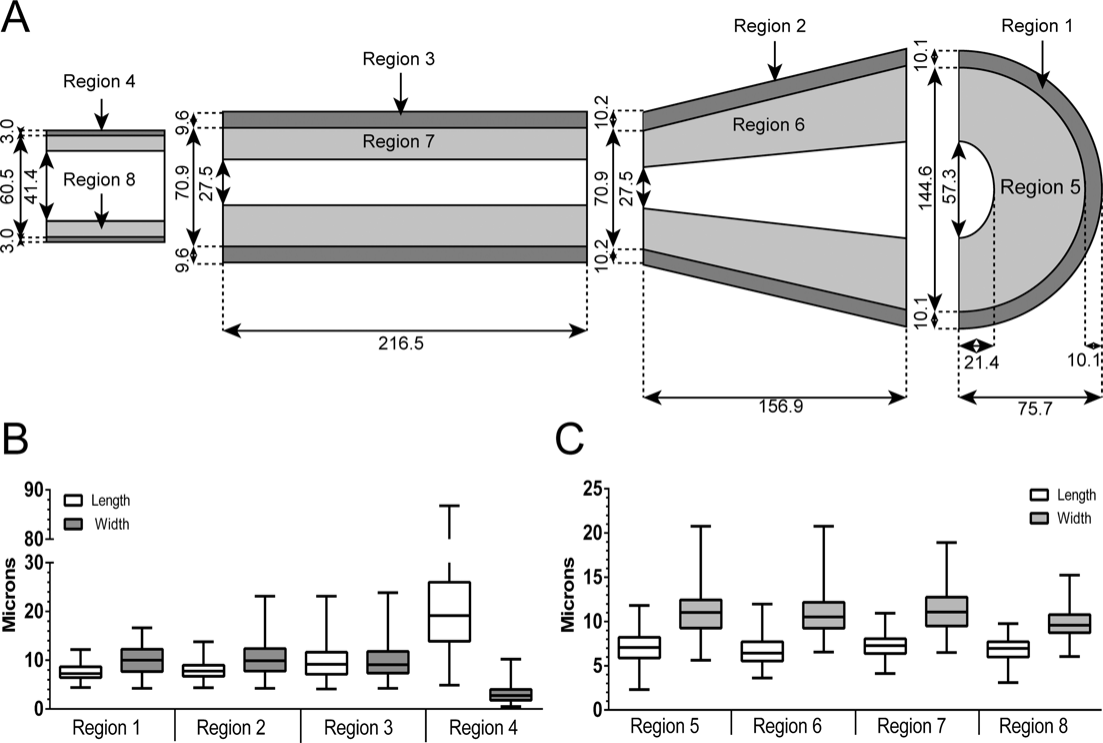
Morphology characterization of the terminal end bud. Glands from 5 and 6 week old mice were embedded, sectioned and stained for epithelial markers and measurements were taken of both cells and the TEB structure. A) Regional measurements of the TEB are represented in a scaled schematic (n values: Regions 1, 2, 5, and 6 = 15 TEBs, Region 3 = 23 TEBs, Region 4 = 8 TEBs, Region 7 layer diameter = 24 TEBs, lumen = 15 TEBs, length = 29 TEBs, Region 8 = 8 ducts). B) Mean cellular dimensions of the cap and myoepithelial cells, Regions 1–4 (whiskers denote range, n values: Region 1 length = 15 TEBs/ 45 cells, Region 1 width = 10 TEBs/ 126 cells. Region 2 length = 15 TEBs/ 56 cells, Region 2 width = 10 TEBs/ 115 cells. Region 3 length = 23 TEBs/ 273 cells, Region 3 width = 9 TEBs/ 36 cells. Region 4 length = 8 TEBs/ 197 cells, Region 4 width = 8 TEBs/ 197 cells). C) Mean cellular dimensions of body and luminal cells, Regions 5–8 (whiskers denote range, n values: Region 5 width/length = 5 TEBs/ 221 cells. Region 6 width/length = 5 TEBs/ 316 cells. Region 7 length/width = 4 TEBs/ 204 cells. Region 8 length/width = 6 ducts/ 301 cells). See also Table A in S1 Text and S2 Fig.

In order to translate the current knowledge and concepts of mammary gland ductal development into a mathematical model for ductal elongation and displacement, we started the modeling process with a description of the time-invariant geometry of TEBs. We defined the stereotypical morphology of a single TEB in a moving frame (i.e. invading through the fat pad with the speed associated with the ductal elongation) and divided the TEB into eight regions (Fig 1A and S1 Fig). Within the TEB structure, each region contains cells (treated as a single population) that are capable of proliferation and apoptosis. We then used a population dynamics model to describe the fate (proliferation or death) of these cells. In order to maintain a constant number of cells in each region, and a constant size of the regions, proliferation in a given region generates a flux of cells from that region into an adjacent one (Eq 1). Ductal elongation is the net result of these regional fluxes: the number of newborn cells in the TEB generates cell fluxes (based on local conservation laws), with deposition of these cells in the mature duct, thus increasing the length of the subtending duct as the TEB invades (S1 Fig). Thus, the net flux from the entire TEB into the mature duct dictates the rate of elongation.

### Compartmental Population Model

We model each region *i* of the TEB as a stationary compartment that contains a time-invariant number of cells and write the corresponding balance of gain (cell proliferation and influxes) and loss (cell death and outfluxes) terms:

**Model 1: Base equation**.

$$\frac{dN_{i}}{dt}=0=+\sum_{l\neq i}\varphi_{l\to i}-\sum_{l\neq i}\varphi_{i\to l}+r_{i}N_{i}-d_{i}N_{i}$$ (1)

*N*_*i*_ : time-invariant cell number in Region *i*

*r*_*i*_ : average population growth (proliferation) rate in Region *i*

*d*_*i*_ : average population death (apoptotic) rate in Region *i*

*φ*_*l*→*i*_, *φ*_*i*→*l*_ : cell influxes (from adjacent regions *l* to *i*) and outfluxes (from *i* to *l*)

### Theoretical Derivation of the Ductal Elongation Rate

The derivation of the ductal elongation rate can be performed by considering the growth of either the outer myoepithelial (basal) layer of the duct (Eq 1 for Regions *i* ∈ {1,2,3}) or the inner (luminal) layer (Eq 1 for Regions *i* ∈ {5,6,7}). For the purposes of the initial model, we assumed that cap cells are entirely responsible for the formation of the myoepithelial layer of the mature duct, and that the body cells are entirely responsible for the formation of the luminal layer of the mature duct. Such assumptions result in the only non-zero fluxes to be *φ*_1→2_, *φ*_2→3_ and *φ*_3→4_ within the basal layer, and *φ*_5→6_, *φ*_6→7_ and *φ*_7→8_ within the luminal layer (S1 Fig). These fluxes correspond to the net production, by unit time, of newborn cells in each region, which result in the net cell fluxes Φ^*bas*^ (Eq 2) corresponding to the elongation of Region 4 (the basal layer) and Φ^*lum*^ (Eq 3) corresponding to the elongation of Region 8 (the luminal layer).

**Cell flux from the TEB to the mature duct**.

- Basal layer:
  $$\Phi^{bas}=\varphi_{3\to 4}=\sum_{i=1,2,3}\left(r_{i}-d_{i}\right)N_{i}$$ (2)
- Luminal layer:
  $$\Phi^{lum}=\varphi_{7\to 8}=\sum_{i=5,6,7}\left(r_{i}-d_{i}\right)N_{i}$$ (3)

Based on the assumption of homeostasis of the mature duct (i.e., apoptosis and proliferation processes balance in Regions 4 and 8), the duct elongates only because cells are generated within the TEB, with time-invariant rates Φ^*bas*^ and Φ^*lum*^ for the basal and luminal layers, respectively. These two values, by definition, must match since the two layers adhere to one another, and one layer does not outpace the other during the process of elongation, as observed experimentally. The ductal elongation rate of each layer is finally found by equalizing the lateral surface area of the mature duct in a 2D cross-section to the surface monolayer covered by *N* adjacent cells and by taking the time derivative of these expressions (Eq 4).

$$2L\left(t\right)=lN\left(t\right)\Rightarrow \lambda =\frac{dL\left(t\right)}{dt}=\frac{l}{2}\;\frac{dN\left(t\right)}{dt}=\frac{l}{2}\;\Phi$$ (4)

*L*(*t*): length of the mature duct

*N*(*t*): cell number along the mature duct

l: length of a single cell

$\Phi =\frac{dN}{dt}$: time-invariant rate of cells provided to the mature duct by the TEB

$\lambda =\frac{dL}{dt}$: ductal elongation rate (for a monolayer)

### 2D Morphological Characterization of Terminal End Buds–Determination of Region Specifications and Cell Size

To determine the morphological characteristics that define the geometric framework of the model, manual measurements of each region and the individual cells within those regions were taken from histological sections of 5–6 week old paraffin embedded terminal end buds (see Methods: Image Analysis and Measurement and Definition of Terminal End Bud Regions). Regional measurements are represented in Fig 1A as a scaled model of the TEB. Cellular dimensions are represented in Fig 1B and 1C as box plots. In Regions 1–4, the cells become thinner (from 10.1μm to 3.0μm) and the intra-nuclear distance increases (from 7.6 to 21.5μm) as they mature in Region 4 (Fig 1B). The cells in Regions 5–8 are relatively the same size (Fig 1C).

Regions 1 and 5 are represented as hollow half-discs and make up the leading tip of the TEB. The total length of these two regions is 85.8μm (75.7+10.1), and the width of these two regions is 164.8μm (144.6+(10.1x2)) (Fig 1A). Regions 2 and 6 are represented as trapezoids, with the larger end joining Regions 1 and 5 and sharing the same total width. The smaller end of Regions 2 and 6 join with Regions 3 and 7 at a total width of 91.3μm (70.9+(10.2x2))(Fig 1A). Regions 3 and 7 maintain approximately this width for a distance of 216.5μm, which we defined as the length of duct refractory to ovarian hormone treatment (S2 Fig). The mature regions of the duct, Regions 4 and 8, have a total width of 66.5μm (60.5+(3.0x2)) (Fig 1A). All measurements are summarized in Table A in S1 Text.

### Determination of Cell Cycle Kinetics and Proliferation Rate

To determine the proliferation rate and cell cycle duration within each of the defined regions of the TEB, we conducted a dual pulse labeling experiment in which proliferating cells were labeled at time zero by incorporation of 5-ethynyl-2-deoxyuridine (EdU). Proliferating cells were subsequently labeled at two hour increments over 24 hours with a 5-bromo-2-deoxyuridine (BrdU) pulse, and glands were harvested two hours after BrdU pulse labeling (see Methods Section: Determination of Proliferation Rates and Cell Cycle Dynamics).

**Fig 2:**
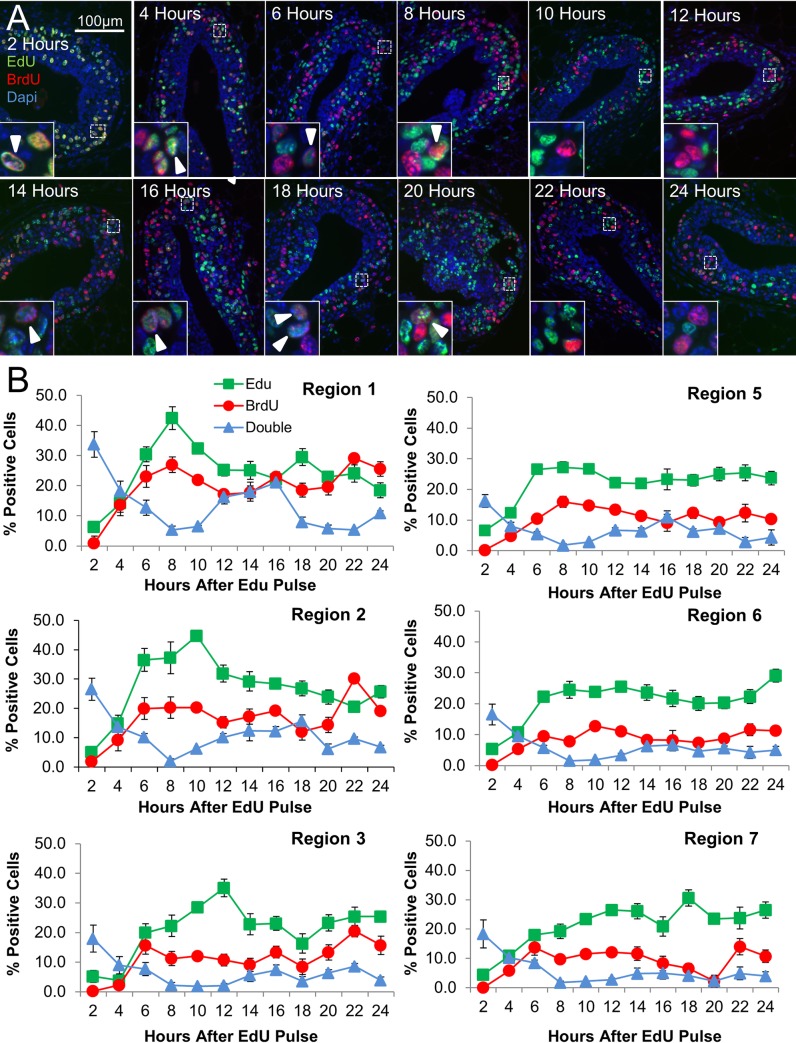
Determination of proliferation rate and cell cycle dynamics. Proliferation rates were analyzed by performing a dual labeling experiment with thymidine analogs EdU and BrdU. Mice were given a pulse of EdU at time “0”, then pulsed with BrdU every 2 hours for 24 hours. Mice were harvested 2 hours after pulsing with BrdU. A) Representative images of TEBs from each time point stained for EdU and BrdU incorporation. B) Quantification of EdU and Brdu single and double-positive populations throughout the time course indicate an S phase duration of 6 hours and total cell cycle time of 16 hours (n = 3 mice, 12 glands, 10 TEBs). See also S3 Fig.

Representative images from each time point are presented in Fig 2A. In all regions, the number of EdU/BrdU double positive cells was highest at time 0, and reached its lowest levels eight hours after EdU labeling, indicating an S -phase duration of about six hours (+/- ~30 minutes). In Regions 1, 2, 3, and 5, the number of double positive cells peaked 16 (Regions 1, 2and 5),18 (Region 3), or 22 (Region 3) hours after EdU labeling, suggesting that cells in each region re-enter the cell cycle. In Regions 6 and 7, the number of double positive cells remained low, suggesting that these cells do not re-enter the cell cycle at high frequency (Fig 2B). Total proliferation levels did not change substantially over time in each region (S3A Fig). Additional time points out to 120 hours were also investigated (S5 Fig). To determine the duration of G2/M phase in the EdU labeled cells, we performed further EdU/BrdU pulse time points at 30 min, 1 hour and 1.5 hours and performed immunofluorescent (IF) staining for phospho-histoneH3 (pHH3) colocalization with EdU. Levels of EdU+pHH3+ cells peaked in all regions two hours after EdU labeling. By eight hours after EdU labeling, cells were no longer co-stained with pHH3. Together with the previous finding that the S phase is ~6 hours long, this indicates a G2/M phase duration of two hours +/- approximately 30 min (S3B and S3C Fig).

### Evaluation of Cell Death—Apoptosis Occurs Primarily in Regions 5, 6, and 7

**Fig 3:**
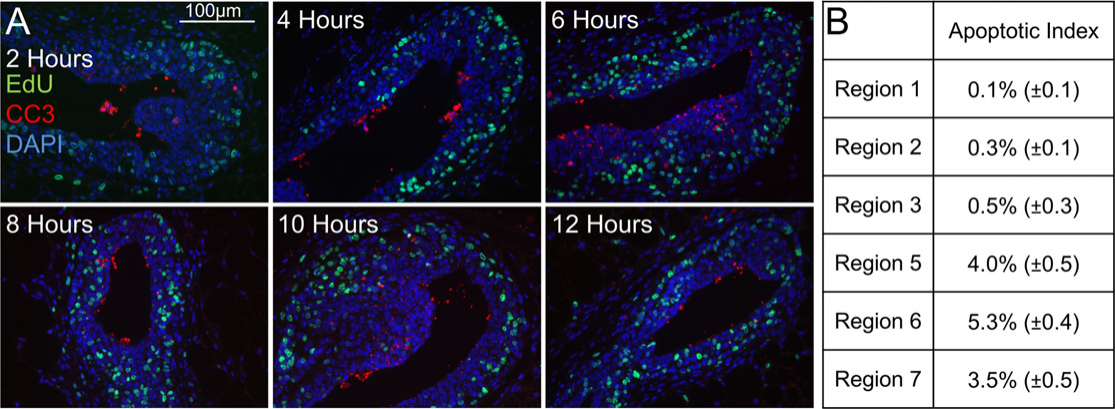
Analysis of apoptosis by Cleaved Caspase III (CC3) staining. Apoptotic levels were determined by cleaved caspase III staining. A) Representative images of TEBs stained for CC3 and EdU incorporation. B) Quantification of CC3 positive cells indicates the majority of apoptosis occurs in Regions 5, 6, and 7 (n = 50 TEBs). See also S4 Fig.

To determine the level of apoptosis occurring within each region of the TEB, we performed IF staining for cleaved caspase III (CC3), a marker of mid-stage apoptosis (See Methods Section: Immunofluorescent Staining Of Tissue). Representative images from each time point are shown in Fig 3A. The majority of apoptosis occurs in the inner regions of the TEB (4.0% ±0.5 in Region 5, 5.3% ±0.4 in Region 6, and 3.5% ±0.5 in Region 7) (Fig 3B). Although the location of apoptosis is consistent with published TUNEL assay data (an indicator of late stage apoptosis) [40], the total levels are ~50% lower probably due to differences in apoptotic stage and the possibility of non-CC3-mediated cell death mechanisms being used. Apoptosis levels in the outer basal layer were less than 1% (0.1% ±0.1 in Region 1, 0.3% ±0.1 in Region 2, and 0.5% ±0.3 in Region 3). Additionally, the time of CC3 positivity after EdU labeling was found to peak between 6 and 10 hours (S4 Fig).

### Initial Model Prediction Indicates Additional Processes Are Necessary to Achieve Model Accuracy

To calculate the rate at which the mammary duct elongates, measurements corresponding to the current scientific knowledge (proliferation and apoptosis rates (S1 Text, section A), cell cycle and apoptosis times, and regional cell numbers (Table B in S1 Text) were used to calculate net population growth rates in the basal regions 1, 2, and 3 and the luminal regions 5, 6, and 7. These growth rates were input into the base mathematical equation (Model 1) (Eqs 2–4). The predicted linear elongation rate for the basal layer was 1.24 (±0.09) mm/day, while the predicted elongation rate for the luminal layer was 0.78 (±0.07) mm/day. This difference indicated that these initial parameters, which encompass much of the known biology of TEB, were not sufficient to model elongation because the results indicate that the two layers do not grow in a coordinated fashion, but rather that they elongate independently, which is not in accordance with observed biology. This result indicated that our model is incomplete.

### Investigation of Cap Cell Behavior Reveals Migration into the Body Cell Layer and Preferential Apoptosis Once There

**Fig 4:**
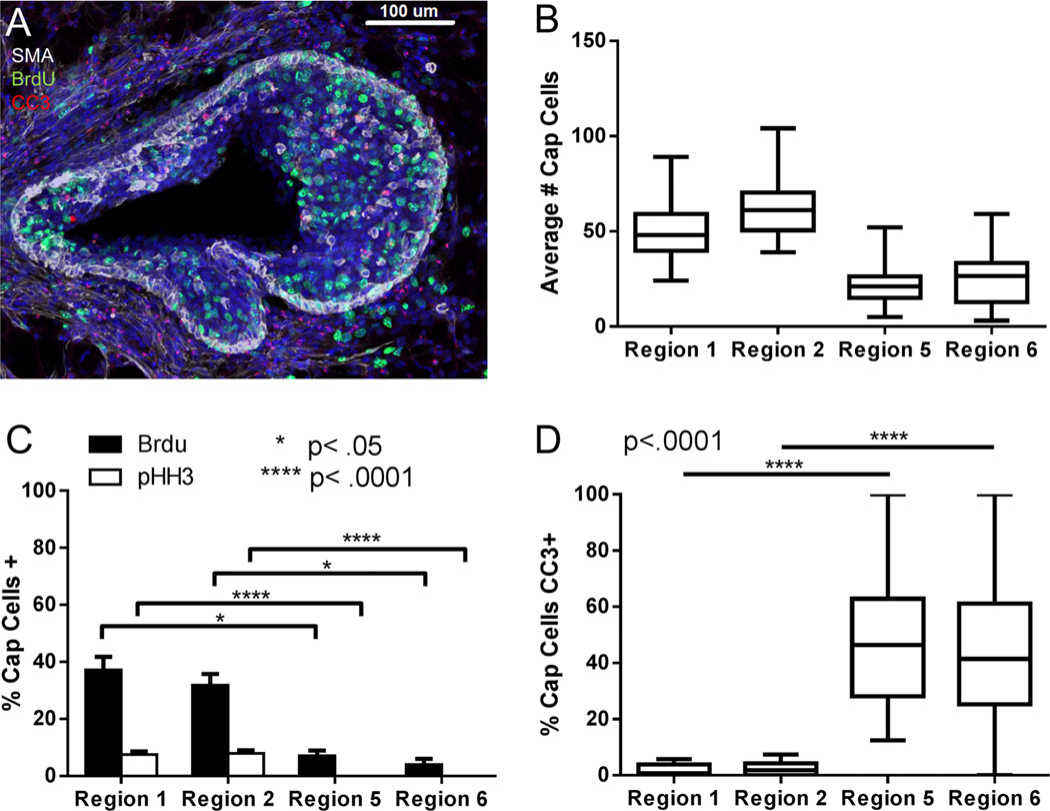
Cap cells migrate into body cell layer and undergo apoptosis. Proliferation status and apoptotic status of SMA+ cells were investigated in both the outer regions (Region 1 and 2) and the inner regions (Regions 5 and 6) of the TEB. A) Representative image of a TEB triple stained for SMA, BrdU, and CC3. B) Mean number of SMA+ cells present in each region, (whiskers denote range) n = 34 TEBs. C) SMA+ cells also positive for BrdU or pHH3 in each region presented as mean ±SEM, *p < .05, ****p < .0001, n = 34 TEBs. D) Quantification of SMA+ cells positive for CC3 in each region presented as mean, ****p<0.0001 (whiskers denote range) n = 34 TEBs.

To address what process(es) were missing from our model, we sought to quantify a previously unappreciated experimental observation that cap cells migrate into the body cell layer [33]. In 1983, Williams and Daniel used time-lapse video to study TEBs and noted that cap cells “migrate from their peripheral location into the deeper regions of the end bud” [33]. Indeed, we found many SMA and p63 positive cells within Regions 5 and 6 (Fig 4A and 4B); however, the fate of these cells was still unknown. This cap cell migration introduces an additional flux of cells from the basal to the luminal regions into the model, and would therefore decrease the predicted elongation rate for the basal layer, while potentially increasing the predicted elongation rate for the luminal layer.

One hypothesis is that migrating cap cells contribute to the body cell layer and may represent mammary multipotent progenitors or stem cells [33, 41]. To address the fate of these cells, we performed staining for SMA and either BrdU or pHH3. Quantification of these images revealed that while SMA positive cells in Regions 1 and 2 have high levels of BrdU incorporation (37.2% ±4.5 and 31.9% ±3.9 respectively) (Fig 4C), SMA positive cells in Regions 5 and 6 had significantly lower levels (7.1% ± 1.8 and 4.0% ± 2.0 respectively, p<0.0001)(Fig 4C), implying that cap cells present in the body cell layer are not as proliferative. Additionally, staining for SMA and pHH3 indicate that cap cells in the body cell layer were not undergoing mitosis (0.0% in Region 5 and 6 versus 7.6% ± 1.0 in Region 1 and 8.0% ± 1.1 in Region 2, p<0.05)(Fig 4C).

To determine if migrating cap cells undergo apoptosis, we stained for SMA and CC3. Quantification of these images found that indeed cap cells present in Regions 5 and 6 were undergoing cell death at a much higher rate than SMA positive cells in Regions 1 and 2 (46.9% ± 4.0 in Region 5 and 42.0% ± 4.1 in Region 6 versus 1.8% ± 0.4 in Region 1 and 2.3% ± 0.4 in Region 2, p<0.0001)(Fig 4D). Overall, these data suggest that cap cells present in the body cell layer are no longer proliferative, and are actively undergoing apoptosis. Thus, they do not appear to contribute substantially to the luminal layer of the mature duct. These results are contrary to the current consensus that cap cells contribute substantially to the luminal lineage. This result also indicated that the mathematical description of cap cell movement from the basal layer into the luminal layer, with subsequent elimination of those cells from the system, was an important missing component to our model.

### Additional Parameters Accounting for Cap Cell Flux and Subsequent Death Adjusts Model Predictions

We accounted for cap cells migrating into the body cell layer by considering a flux of cells *φ*_*bas*→*lum*_ (= *φ*_1→5_ + *φ*_2→6_ + *φ*_3→7_, see S1 Fig), which leads to the modified fluxes Φ^*bas*^ − *φ*_*bas*→*lum*_ and Φ^*lum*^ + *φ*_*bas*→*lum*_ to be considered for the evaluation of the basal and luminal elongation rates (Model 2), respectively (S1 Text, section B). When cap cell specific death is accounted for, our model revealed that the apoptotic index experimentally measured for the luminal layer is underestimated, resulting in inconsistencies in the model. To address this issue, an apoptotic correction factor (*δ* = 97%) was introduced (S1 Text, section C and Table C) into the equation for the luminal layer (Model 3), yielding an apoptotic index of 8.5%. Inclusion of the additional flux and apoptotic correction factor into our equations increased the predicted elongation rate for the luminal layer from 0.78 to 0.81 (±0.08) mm/day, and decreased the rate for the basal layer from 1.24 mm/day to 0.76 (±0.12) mm/day, bringing these two calculated elongation rates much closer together in accordance with the observation that the layers elongate in a coordinated fashion. To validate the accuracy of our prediction we sought to measure ductal elongation *in vivo*.

### Predicted Elongation Rate Does Not Equal the TEB Displacement Rate of 0.54mm per Day

**Fig 5:**
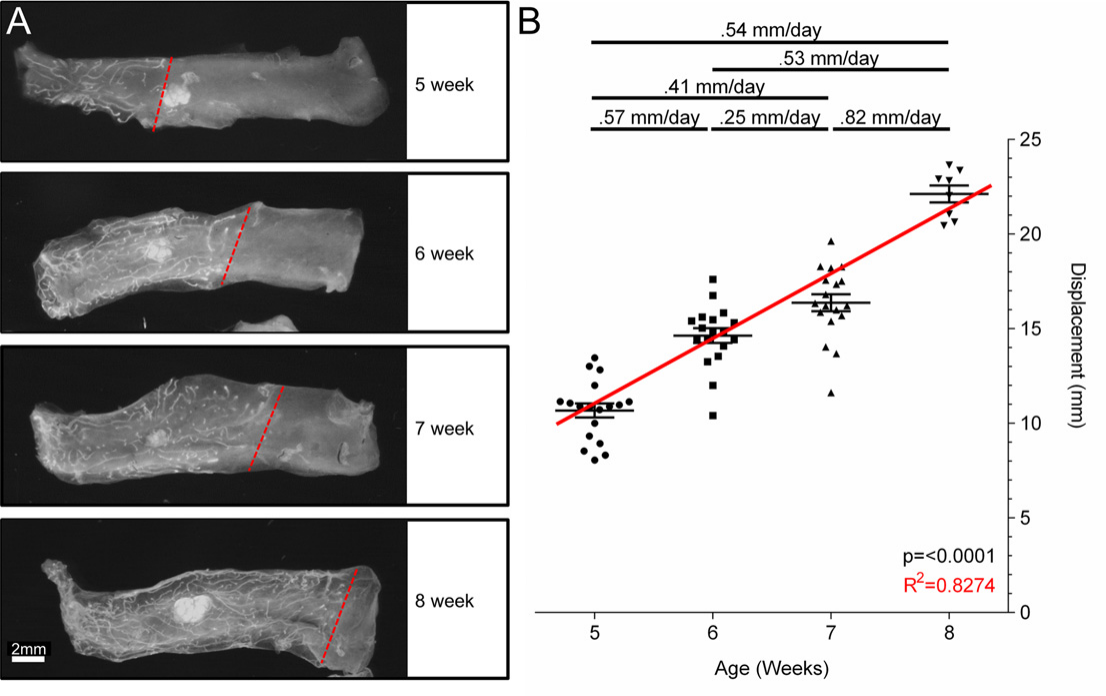
Experimental determination of TEB displacement rate. Quantification of the distance from the nipple to the ductal boundary in 5 (n = 18), 6 (n = 18), 7 (n = 18), and 8 (n = 8) week FVB mice. A) Representative inguinal glands from 5, 6, 7 and 8 week old FVB mice are pictured with a dashed line demarcating the ductal front. B) Quantification of the outgrowths at each time point are fitted with a best fit line (one-way ANOVA p<0.0001, R2 = .8274). On average during puberty, the duct grows at a rate of 0.54 mm per day.

To validate the model prediction that the gland elongates at a rate in the range of 0.76–0.81 mm per day, we measured the rate at which the TEBs are displaced during pubertal elongation *in vivo*. To do this we measured outgrowths in both inguinal glands of FVB mice from the nipple to the ductal front. The length of ductal outgrowth was then compared across each time point to provide the TEB displacement rate (Fig 5). The rate of displacement from weeks 5 to 6 (0.57mm/day), 6 to 7 (0.25mm/day), and 7 to 8 (0.82mm/day) averages 0.54 mm/day over the three week period, a rate significantly less than the predicted elongation rate of 0.76–0.81mm/day.

Our model prediction represents the linear elongation rate of a new duct formed as a straight tube (or the speed at which the new duct forms in a straight line), which is different from the displacement rate that we measure here. The displacement rate is not a measure of the total length of duct, but rather it is based on the shortest distance between the nipple and the ductal front and does not take into account the actual path (i.e. turning) of the TEB. In order to compare our mathematical elongation rate prediction to the measured displacement rate, we must convert the linear output to its displacement equivalent.

### TEB Bifurcation and Turning Contributes to Path Tortuosity

**Fig 6:**
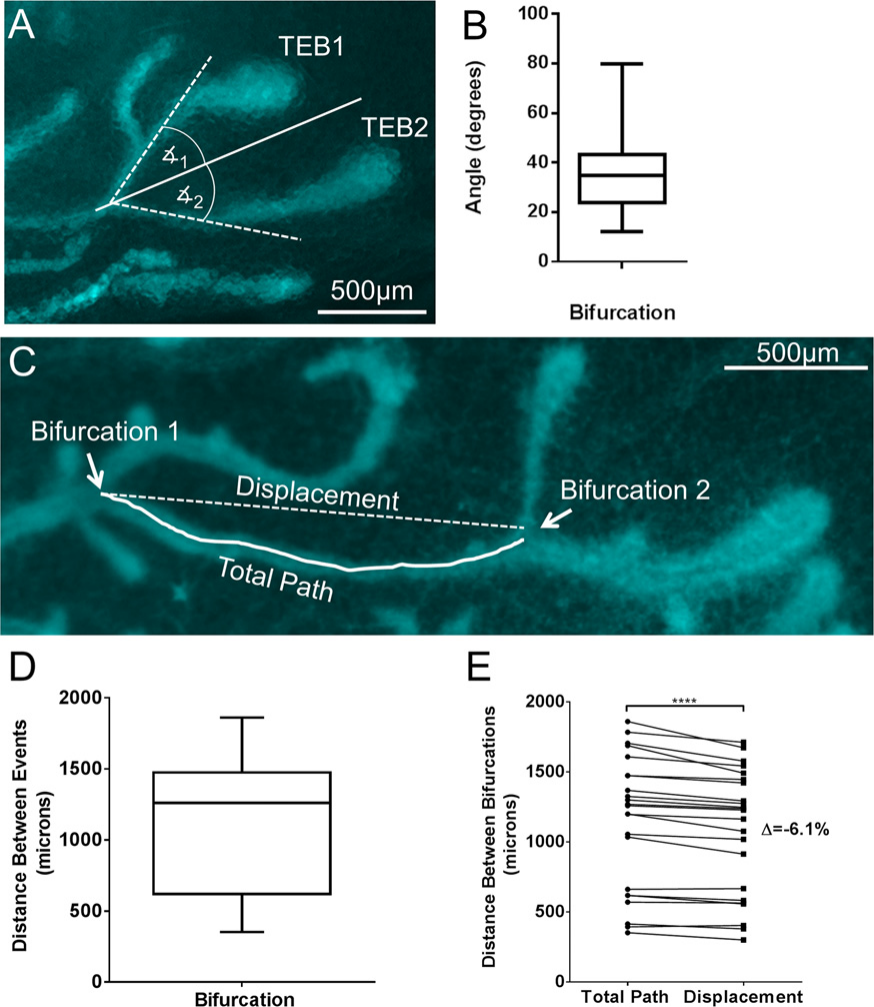
Assessment of TEB path tortuosity. The angle of deflection caused by bifurcation, the frequency of bifurcation, and the disparity between displacement and path tracing measurements were measured. A) Representative image of a TEB bifurcation event with the original path of the TEB noted as a solid line, the new path of each TEB as a result of the bifurcation are noted as dotted lines and the angles measured noted. B) Quantification of the angle of deflection presented as mean (whiskers denote range)(n = 62). C) Representative image of TEB’s growth path with 2 bifurcation events noted and the length of duct between noted with a solid line. D) Quantification of the total length of duct between bifurcation events is presented as mean (whiskers denote range)(n = 23). E) Total length of duct measurements were compared to corresponding displacement measurements. Displacement measurements consistently underestimated the total length by 6.1% (±0.9) (p = .0001, paired ratios t-Test, n = 23).

Tortuosity refers to the characteristic of a winding or twisting path as opposed to a straight path. The path of the TEB is tortuous due to both bifurcation events and natural turning. Because bifurcation is a frequent event during elongation, we sought to determine the effect that bifurcation has on the straight line route of a TEB’s growth path. We measured the angle at which a TEB is deflected off its path at the time of bifurcation by measuring the angle between the original path and the path after bifurcation (Fig 6A)(see Methods: Image Analysis and Measurement). We found that the average angle of deflection is 35.5° (±1.9°, n = 62)(Fig 6B). In addition, we evaluated the frequency of bifurcation during pubertal outgrowth. This was determined by measuring the distance (path length) between bifurcation events (Fig 6C). The average distance between bifurcation forks was 1141μm (±99.1, n = 23)(Fig 6D). Additionally, we compared path tracing measurements to corresponding displacement measurements between bifurcation events and found that the displacement measurements consistently underestimate the total distance by 6.1% (±0.9%, n = 23) (Fig 6E).

### Modelling Demonstrates a Significant Effect of Tortuosity on Displacement Measurement

We used the measurements of TEB path tortuosity to convert (S1 Text, section D) our previously predicted linear elongation rates (0.81 and 0.76 mm/day) into the measured displacement rate using our ultimate model (Model 4) (Eqs 5–6) which accounts for the cap cells dropping into the body cell layer (S1 Text, section D):

**Model 4.**

$$\lambda^{bas}=\frac{l^{bas}}{2T}\left(\sum_{i=1,2,3}\left(r_{i}-d_{i}\right)N_{i}-\varphi_{bas\to lum}\right)$$ (5)

$$\lambda^{lum}=\frac{l^{lum}}{2T}\left(\sum_{i=5,6,7}\left(r_{i}-d_{i}\left(1+\delta \right)\right)N_{i}+\varphi_{bas\to lum}\right)$$ (6)

where,

- *φ*_*bas*→*lum*_: flux of cap cells dropping into the body cell layer (Model 2);
- *δ*: correction factor for apoptotic index in the luminal regions (Model 3);
- *T*: average tortuosity of a single duct.

Tortuosity due to bifurcation (a factor of 1.23) and tortuosity due to turning (a factor of 1.06) are calculated using measurements determined in the previous experiment (S1 Text, section D). The predicted rate is converted to displacement rate by dividing our previous predictions of 0.81 and 0.76 mm/day by the total tortuosity (*T* = 1.31). This conversion gives a displacement rate of 0.62mm/day and 0.58mm/day for the luminal and basal layers, respectively (Table D in S1 Text). The difference between the experimentally measured displacement rate of 0.54mm/day and our model’s prediction indicate that these parameters, including the addition of a novel cap cell flux with the luminal apoptotic correction factor, are sufficient to account for the kinetics of ductal elongation. These results also indicate that displacement measurements can underestimate total duct length by 24%.

### Model Iteration Summary and Fit Analysis

**Fig 7:**
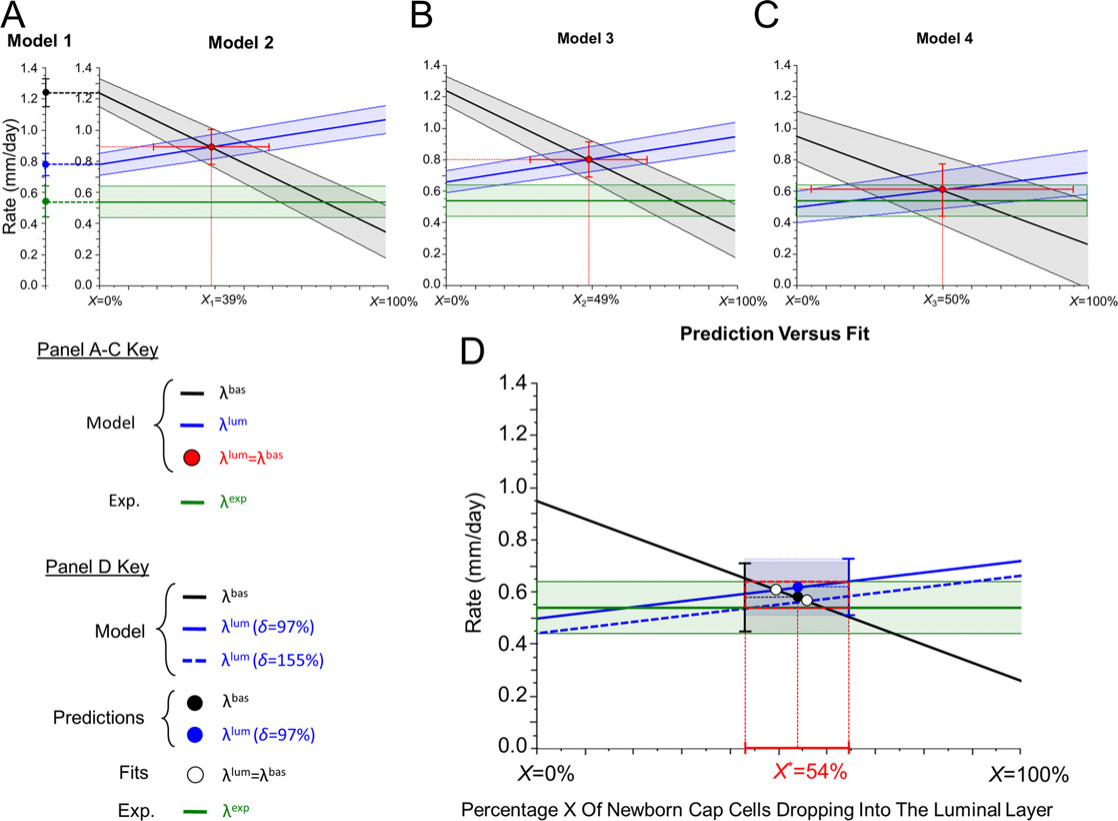
Model iteration summary and fit versus data-informed results. Comparisons between mathematically derived cap cell flux values and values provided by the basal and luminal elongation rates matching condition in the context of the apoptotic correction factor. For each panel green lines represent the experimentally measured rate, black lines represent basal elongation predicted rates and blue lines represent luminal predicted rates. Each bold prediction line is shaded with standard deviation (experimental value) or uncertainty values (model predictions). Model 1 is the “base model”. Model 2 is the “base model + cap cell flux”. Model 3 is the “base model + cap cell flux + apoptotic correction”. Model 4 is the “base model +cap cell flux +apoptotic correction + displacement conversion”. A) Model 1 and 2 outputs are shown. Model 1 predicts two separate rates (no cap cell flux, i.e. *X* = 0%) that both lie outside the experimental dispersion, whereas Model 2 rates predict coordinated growth (for cap cell flux value *X*_1_ = 39%) but a prediction (in red) outside the experimental value error. B) Results from Model 3 which included the required apoptotic correction factor (*δ* = 0.97) for the luminal layer are shown. Matching condition yields a cap cell flux value of 49% (*X*_2_ = 49%) however the predicted rate (in red) still lies outside the experimental dispersion. C) Results from Model 4 are shown after the rates are converted to a displacement rate and *δ* = 0.97. Matching condition yields a cap cell flux of 50% (*X*_3_ = 50%) and the predicted rate (in red) lies within the experimental value. D) A summary of the final model predictions and experimentally measured values are shown. Matching condition values (white dots for minimal value *δ* = 0.97 and for value *δ* = 1.55 corresponding to Humphreys et al) and experimentally based model prediction (blue and black dots corresponding to X* = 54%) cluster in a small range of admissible values compatible with the experimentally measured rate.

Our initial model, Model 1, yielded single value predictions for the luminal and basal layers that were incompatible with known biology, and with our experimentally measured displacement rate of 0.54mm per day (Model 1—Fig 7A). With the addition of the cap cell flux (Model 2—Fig 7A) the model outputs become dependent on the value of this flux, which then allows us to perform a model fit analysis: for a fixed value of *δ*, *φ*_*bas*→*lum*_ (and therefore, the corresponding cap cell fraction *X*, see S1 Text, section E) can be determined by using the matching condition λ^*bas*^ = λ^*lum*^. Without accounting for cap cell specific death (*δ* = 0), the fit predicts an elongation rate that is incompatible with our experimentally measured rate (Model 2—Fig 7A). However, addition of the minimal apoptotic correction factor of *δ* = 0.97 (S1 Text, section C) yielded a cap cell flux value (corresponding to *X* = *X*_2_ = 49%) leading to an elongation rate still outside the error of our experimentally derived levels (Model 3—Fig 7B). When we account for tortuosity, the linear elongation rate is converted to a displacement rate (Model 4—Fig 7C) and we find agreement between the model fitting and our experimentally measured rate, however with a very large range of admissible values of the cap cell flux (Fig 7C red error bar). The ultimate model, when constrained by specific values estimated experimentally (Model 4—Fig 7D) for *X* = X* = 54% and for *δ* = 0.97 (S1 Text, section E), yields values (λ^*lum*^ = 0.62*mm* / *day* and λ^*bas*^ = 0.58*mm* / *day*, Fig 7D blue and black dots) that fall within the error of the experimentally measured displacement rate. When we compare these values to those found from model fitting with *δ* = 0.97 (Fig 7D blue solid line and white dot) we also find a rate within this error box. Finally, when we apply an apoptotic correction factor (*δ* = 1.55) equivalent to the 11% apoptotic index reported by Humphreys et al [40] (Fig 7D blue dotted line), we find a flux value by model fitting that exactly matches for both TEB layers (white dot), and lies within the same error box. These results illustrate that our final model predictions, based on experimentally estimated values of *X* and *δ*, provides values of the elongation rates that 1) lie within the error range of our measurement and 2) are consistent with values forced by fitting but ignoring the biological cause, thus strengthening our final model and our modeling approach’s validity.

## Discussion

The TEB is the driving force for mammary duct invasion and elongation into the fat pad. Here, we exploited the geometric constraints on TEB size to develop a mathematical model for ductal elongation and displacement. This iterative modeling approach identified a set of experimentally measurable parameters required for accurate modeling. Our base model (Model 1) incorporating only region-specific cell number, proliferation rate (BrdU) and apoptotic index (CC3), predicted two different elongation rates for the basal and luminal layers. This prediction was inconsistent with the biological observation that the two cellular compartments grow in a coordinated fashion, and required identification and evaluation of additional processes and parameters. Parameters were added sequentially to the model until the predicted elongation rates of the two cellular compartments could be matched (Models 2 and 3), thereby demonstrating that our investigative approach of ductal elongation helped identify the major biological processes involved, similar to the approach by Hoehme et al. [19] in the context of liver regeneration. Once the predicted elongation rate was established, conversion to an observed displacement rate (Model 4) required measurement of average branch angle and duct tortuosity. The results from this process have a significant impact on how developmental phenotypes in elongating structures are interpreted generally, as well as for our understanding of cellular dynamics within the TEB.

Cap cells have long been referred to as putative reservoir for mammary stem cells, and have been credited with contributing to both the basal and luminal lineages [33, 38, 42, 43]. In 1983, Williams and Daniel described time lapse video of cap cells migrating into the body cell layer and out of view. They hypothesized that cap cells that migrate into the body cell layer differentiate into luminal cells, and that they are examples of multipotent progenitor cells [33]. An alternative hypothesis is that migrating cap cells rejoin the basal layer as the duct elongates. Numerous microscopy analyses have shown the presence of cap cells in the body cell layer [44–46]; however, none of these studies have investigated the fate of cap cells once actually there. More recent lineage tracing experiments have led to conflicting conclusions about the contribution of cap cells to the luminal lineage. The most recent studies by Rios et al maintained the historical view that cap cells in the TEB give rise to luminal cells [36, 37]. However their lineage tracing data did not address whether this contribution is due to migration of cap cells into the body cell layer, or due to asymmetric division directly from the cap cell layer into the body cell layer.

The failure of Model 1 along with the conflicting positions on cap cell biology led us to investigate Williams and Daniel’s observation further. We demonstrate that the migrating cap cells are no longer proliferative by both decreased BrdU labelling and low pHH3, and in fact, are actively undergoing programmed cell death at a high rate. Using the measurements of proliferation and the number of cap cells present in the body cell layer, we were able to calculate the percentage of newborn cap cells that migrate into the body cell layer to die. If we assume that cap cells are dropping in from only Regions 1 and 2 we calculate that a striking 54% of the daughter cells actually migrate into the body cell layer to undergo apoptosis (39% if region 3 is included 3)(S1 Text, section E), and thus are not used productively for duct formation. Together with recent lineage tracing data [37], these data strongly suggests that few, if any, cap cells contribute to the body cell layer, or rejoin the basal layer.

The high levels of CC3+ cap cells present in the body cell layer forced us to reexamine the dynamics within the body cell layer and adjust the mathematical model. Although several groups have previously investigated cell death in the body cell layer and accepted it as a major mechanism of lumen formation [40, 44, 47, 48], none have confirmed the lineage of these dying cells. Our studies have shown the majority of CC3+ cells to be of cap cell origin, leaving only a small percentage of true body cells undergoing caspase-3-mediated cell death. When this phenomenon was introduced into the mathematical model in Model 3, a correction factor of 97% was needed for the luminal layer in order to keep the mathematical model consistent (S1 Text, section C). This correction factor represents an underestimation of total cell death by CC3 positivity. These model manipulations provide mathematical evidence that true body cell clearance levels are higher than our biological measurements have indicated.

Given our results, it is possible that CC3-mediated apoptosis is a mechanism for removal of cap cells specifically, rather than a mechanism of luminal cell clearance. Previous work indicated that cap cells, when detached from the cap cell layer, undergo apoptosis in response to anoikis. In Netrin-1 KO mice, unattached cap cells die at a rate 17-fold higher than attached cap cells. This coincides with a 2-fold increase in the number of cap cells present in the body cell layer [49]. Additionally, targeted disruption of BIM, a pro-apoptotic factor, leads to a 5.6-fold increase in p63 positive cells in the TEB [44]. It remains unclear whether non-CC3-mediated cell death mechanisms in the body cell layer may play a role in lumen formation.

In our ultimate model, Model 4, the net increase in cell number, cell migration, and the geometric constraints within the TEB creates cellular fluxes into the mature duct, which results in forward movement of the TEB as it elongates through the fat pad. However, the ultimate displacement within the fat pad is modulated by changes in direction due to branching and turning (tortuosity). The behavior of our final model has illustrated a significant problem with regard to the use of traditional measurements and terminology for characterizing developmental changes.

Mammary gland researchers tend to describe mutant developmental phenotypes as ‘delayed’ or ‘promiscuous’ when they see a decrease or increase, respectively, in the distance from the nipple (or lymph node) to the ductal front (a direct measurement of displacement), or in the percent fat pad filled (an indirect measurement of displacement) [50–52]. This is misleading language born from a failure to make the distinction between elongation rate and displacement rate and leads to a misunderstanding of the true nature of ductal development. As demonstrated by our measurements and mathematical model, the total distance a TEB must traverse as the duct elongates is significantly greater (~24%) than a traditional displacement measurement would suggest. For example, in the Plk2 knockout (KO) mouse, a “delay” and “impairment” in ductal elongation was reported, with Plk2 KO glands filling the fat pads 2 weeks later than WT mice. However, increased proliferation and hyperbranching were also observed concomitant with the elongation “delay” [50]. In light of our demonstration that bifurcation decreases the measured displacement by 19%, it is not surprising that glands with increased branching take longer to fill the fat pad. In fact, this has been observed in a number of other mouse models with both gain- and loss-of-function mutations usually involving growth factor signaling [53, 54]. While similar phenotypes have traditionally been labeled as developmental delays, they actually could represent a completely different kind of alteration in ductal development. The precise characterization of these phenomena will require a more careful analysis of the kinetics of ductal morphogenesis.

In its current state, our model has some limitations. The first is that the current model only allows for changes in histology (cellular number and arrangements) not gross morphology (increased branching). This is problematic because increased proliferation is frequently coupled with increased branching in the animal. The ultimate goal of this project is to create a multi-scale model which functions at different levels of development, from histology (current model) to cellular behavior and signaling, and gross morphology, which will address these limitations. A second limitation is that the parameters were evaluated as averages and assumed to be constant over time, while variations in these values could change the predicted elongation rate and may account for the natural variation in growth rates (Fig 5). We wish to note that our model is fully integrative, in the sense that it has not been ‘fit’ to the data, nor does it have degrees of freedom within the parameters, rather the data have been used exactly to inform the model. However, we were able to demonstrate that fitting our model to previously published data does validate our experimentally measured parameters, at least in the context of apoptotic index and cap cell migration (Fig 7).

Because of the natural genetic variation in mammary ductal development it will be of interest to apply this mathematical model to other mouse strains that exhibit markedly different kinetics of ductal morphogenesis [55]. This model could also be adapted for modeling other branching organs, such as the salivary gland, lung, and prostate. In addition, because the TEB shares several important features with tumors (heterogeneous cell populations, high proliferation levels, invasive behavior, and angiogenic properties), this model should also provide an ideal ground state for modeling and investigating ductal carcinoma in situ (DCIS) development as well as growth and behavior of invasive cancers in the breast.

## Methods

### Mouse Strains Used

FVB/NJ and FVB.Cg-Tg(ACTB-EGFP)B5Nagy/J mice were used for all experiments except to study cap cell migration into the body cell layer, for which Balb/C mice were used. All animal work was performed according to IACUC approved protocols.

### Evaluation of Ductal Growth Rate

Female FVB virgin mice were harvested at exactly five, six, seven, and eight weeks of age. Total fat pad length was measured prior to dissection. Glands were harvested and fixed in 4% Paraformaldehyde for 3 hours and cleared in 50% Glycerol 50% PBS solution overnight. Both inguinal glands were stained with DAPI according to the protocol previously described [56]. Images were taken using a Leica MZ16F dissecting microscope equipped with a Leica DFC300 FX camera and analyzed with MetaMorph 7.1 (Molecular Devices Inc.). The total length of the fat pad and the distance from the nipple to the ductal front were measured using MetaMorph 7.1 (Molecular Devices Inc.). The true distance of ductal growth was calculated by multiplying the percent fat pad filled by the original length of the fat pad prior to harvest.

### 2D Rendering of the TEB

A 2D schematic of a TEB medial longitudinal section was generated based on experimentally measured histological and morphological features of a typical TEB. Outer and inner compartments (myoepithelial and luminal) were designated as separate, then each was divided into regions based on morphology.

### Determination of Proliferation Rates and Cell Cycle Dynamics

To evaluate proliferation rates and cell cycle parameters, we conducted a dual pulse cell labeling experiment [57]. Five to six week old mice were pulsed with 50μg/ body weight EdU (25mg/ml in PBS) by intraperitoneal injection at time 0. An injection of 2mg/g body weight BrdU (25mg/ml in PBS) was given to cohorts of mice in two hour increments after time 0 for a total of 24 hours. (Additional long term pulse experiments were conducted with EdU given to all mice at time 0 and BrdU given at 24 hours increments for 120 hrs.) Glands were harvested two hours after receiving the BrdU pulse (3 animals per time point). Glands were fixed in 4% PFA for 3 hours and stored in 70% EtOH before paraffin embedding. EdU was visualized using the Invitrogen Click-iT Kit. All images were counted manually.

### Immunofluorescent Staining of Tissue

Harvested glands were fixed in 4% paraformaldehyde for 3 hours and then stored in 70% EtOH. Samples were embedded in paraffin and serially sectioned. Sections were deparaffinized in NeoClear and decreasing concentrations of EtOH (100%, 95%, 80%, and 70%). Antigen retrieval was accomplished by incubating slides in citrate buffer pH6 in a pressure cooker. Slides were cooled in water and washed in TBST before staining. Slides were blocked in 10% normal goat serum and MOM IgG blocker for 1 hour at room temperature. The different primary antibody combinations were incubated over night at 4°C. Sections were rinsed for 10 min in TBST and incubated with secondary antibodies 1:500 at room temperature for 1 hour. The following primary antibodies were used: anti-BrdU (Invitrogen B35128), anti-phospho Histone H3 (Milipore 06–570), anti-Cleaved Caspase III (Cell Signaling 9961s (D175)) anti-p63, anti-Cytokeratin 5 (AbCam ab52635), anti-Smooth Muscle Actin (Sigma Aldrich A2547). The CK8 monoclonal antibody developed by Phillippe Brulet and Rolf Kemler was obtained from the Developmental Studies Hybridoma Bank, created by the NICD of the NIH and maintained at The University of Iowa, Department of Biology, Iowa City, IA 52242. The following secondary antibodies were used: anti-mouse Alexa Fluor 594 (Invitrogen A11005), and anti-rabbit Alexa Fluor 594 (Invitrogen A11012). Nuclei were stained with Dapi and slides were mounted using Vectashield (Vector Laboratories).

### Image Analysis and Measurement

All measurements were done manually because automated methods do not perform well with histological sections of mammary tissue due to complex image regions/scenarios (i.e. cells close together, apoptotic clumps of debris, multiple foci within one nucleus). Further, all measurements within a given experiment were done by a single individual in order to eliminate variation in methodology from individual to individual. All methods of measurements were reviewed for consistency and spot-verified by a second individual, as well as by the senior co-authors, over the course of the study. All raw images are available from the corresponding author upon request.

The medial longitudinal section of each TEB was selected from serial sections and imaged. Imaged TEBs were broken down into regions 1–8 manually, and cells counted manually based on staining combinations. Sections were imaged using a Zeiss Axioskop 2 Plus fluorescent microscope equipped with an AxioCam MRm camera. Regional and cellular dimensions were measured using Zeiss Axiovision 4.8 software. Whole mount images were taken with a Leica MZ16F dissecting microscope equipped with a Leica DFC300 FX camera and analyzed with MetaMorph 7.1 (Molecular Devices Inc.). Bifurcations were chosen when the TEBs could be traced back to the bifurcation event. The growth path just prior to bifurcation was traced, and the subsequent angles off of that path by each new TEB were measured using the Metamorph 7.1 (Fig 6A).

### Definition of Terminal End Bud Regions

TEB regions were defined by morphological cues. A line drawn across a longitudinal histological section of a TEB at the widest point will divide Regions 1 and 5 from Regions 2 and 6. The Regions forward of the line are Region 1 (a single cell outer most layer) and Region 5 (which includes all cells between the lumen and the outer single layer). A line across the duct at the neck of the TEB will divide Regions 2 and 6 from Regions 3 and 7. This line should be drawn at the location where the narrowing stops (after which the duct becomes a uniform thickness). This length can vary between TEBs and an average value is presented in Fig 1. Region 2 is the single cell outer layer within this length and Region 6 contains all the cells between the lumen and the single cell outer layer within this length. Regions 3 and 7 are defined by the boundary between TEB-proximal ductal cells lacking differentiation capacity when exposed to pregnancy hormones relative to more distal ductal cells that are capable of morphological differentiation in response to hormones, as demonstrated in S2 Fig. In our experiments with FVB mice, the length of these two regions is 216.48μm. Region 3 is the single cell outer layer within this length and Region7 contains all cells between the lumen and the single cell outer layer. Regions 4 and 8 are mature duct distant to the TEB and were measured from sections not containing TEB features.

## Supporting Information

S1 FigSchematic of Cellular Fluxes and Regions in the TEB.The TEB is divided into regions based on morphology. The outer cap cell and myoepithelial layer is designated as regions 1–4. The inner body cell and luminal layer is designated as regions 5–8. Each region has independent outflux values resulting from net proliferation and yielding to total fluxes toward the mature duct. Non-zero fluxes included in the Base Model (Model 1) are shown in red, additional basal to luminal fluxes included in the subsequent models (Models 2–4) are depicted in grey. The rate at which the duct elongates is determined by the total flux and the length (l) of a mature myoepithelial cell in 2D.(TIF)

S2 Fig Length of Hormone-Refractory Region of the Immature Duct. 5–6 week old mice were treated with estrogen and progesterone (E+P) in sesame oil or oil only for 10 days in order to induce alveologenesis in mature alveolar cells. The distances from the TEB to the first branchpoint or alveolar bud were measured. A) Representative image of a control treated gland with an arrow head indicating a branch point. B) Representative image of an E+P treated gland with an arrow indicating an alveolar bud. C) Quantification of the average distance between the TEB and branch/bud event as mean ±SEM; Control mean = 636.7μm ±59.28 n = 26, E+P mean = 216.48μm ±13.47 (p < .0001) n = 29.
(TIF)

S3 Fig Total Proliferation and G2/M Phase Duration. All mice were given a pulse of Edu at time 0 and BrdU two hours before harvest. Additional time points of 30 min, 1 hour and 1.5 hours was used for G2/M phase analysis. A) Total levels of BrdU are constant over time (each time point is mean ±SEM). B) Representative images of pHH3 and EdU co-staining. C) Quantification of EdU/pHH3 double positive cells by region over 12 hours indicate that duration of G2/M phase is 2 hours (each time point represents mean ±SEM, n = 10 TEBs per time point).
(TIF)

S4 Fig Apoptotic Time. EdU pulsed cells were stained for CC3 to determine time to apoptosis. Quantification of EdU/CC3 double positive cells (mean ±SEM, n = 7–10 TEBs) indicates that most cells undergo apoptosis by 12 hours after EdU labelling.
(TIF)

S5 Fig Long Term EdU Pulse. Mice were given a pulse of EdU at time 0 and glands were harvested every 24 hours (with a BrdU pulse given 2 hours before harvest) for up to 120 hours. A) Representative images of TEBs are shown including time points from previous pulse (2 and 12 hours). B) EdU and BrdU single and double positive cells were quantified by region up to 120 hours after EdU pulse (mean ±SEM, n = 7–10 TEBs).
(TIF)

S1 Text Summary Tables and Mathematical Derivations. Tables of measurement values, experimental values, input parameter values, summary of predictions, and cell number by region validations are presented in tables A-E. Further explanations of mathematical derivations for A) Converting cell fractions into rates, B) Modeling cap cells dropping into the body cell layer, C) Correction factor for apoptosis rates in the body cell layer, D) Conversion from elongation to displacement rate, and E) General Formulation, parameters and validation are presented in the text.
(DOCX)
