# Supplementary figures and images for: A Geometrically-Constrained Mathematical Model of Mammary Gland Ductal Elongation Reveals Novel Cellular Dynamics within the Terminal End Bud
Source: PLoS Comput Biol. 2016 Apr 26;12(4):e1004839. doi: 10.1371/journal.pcbi.1004839 (PMC4845990; doi:10.1371/journal.pcbi.1004839)

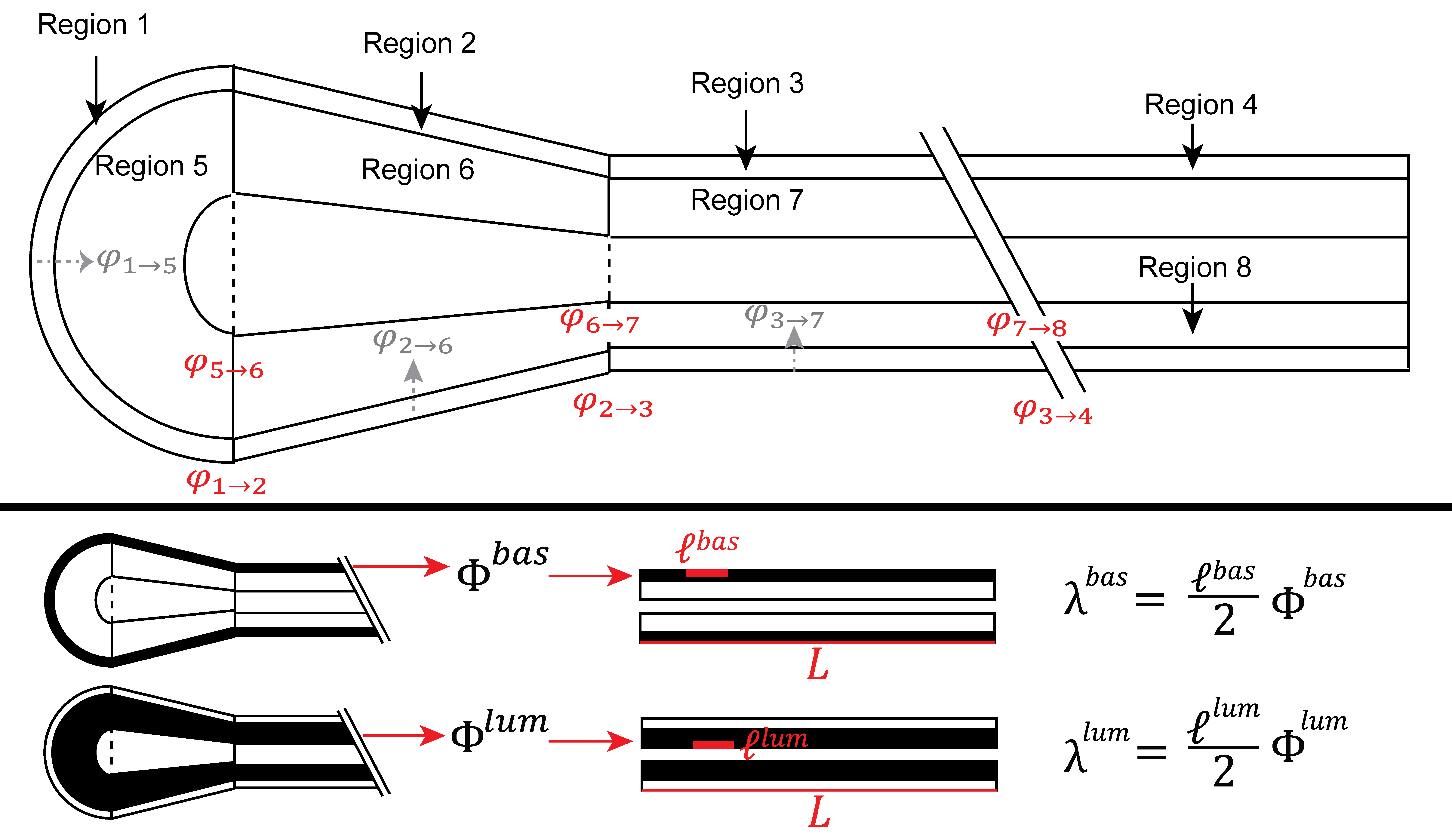

Supplement: S1 Fig — The TEB is divided into regions based on morphology. The outer cap cell and myoepithelial layer is designated as regions 1–4. The inner body cell and luminal layer is designated as regions 5–8. Each region has independent outflux values resulting from net proliferation and yielding to total fluxes toward the mature duct. Non-zero fluxes included in the Base Model (Model 1) are shown in red, additional basal to luminal fluxes included in the subsequent models (Models 2–4) are depicted in grey. The rate at which the duct elongates is determined by the total flux and the length (l) of a mature myoepithelial cell in 2D. (TIF) [file pcbi.1004839.s001.tif]

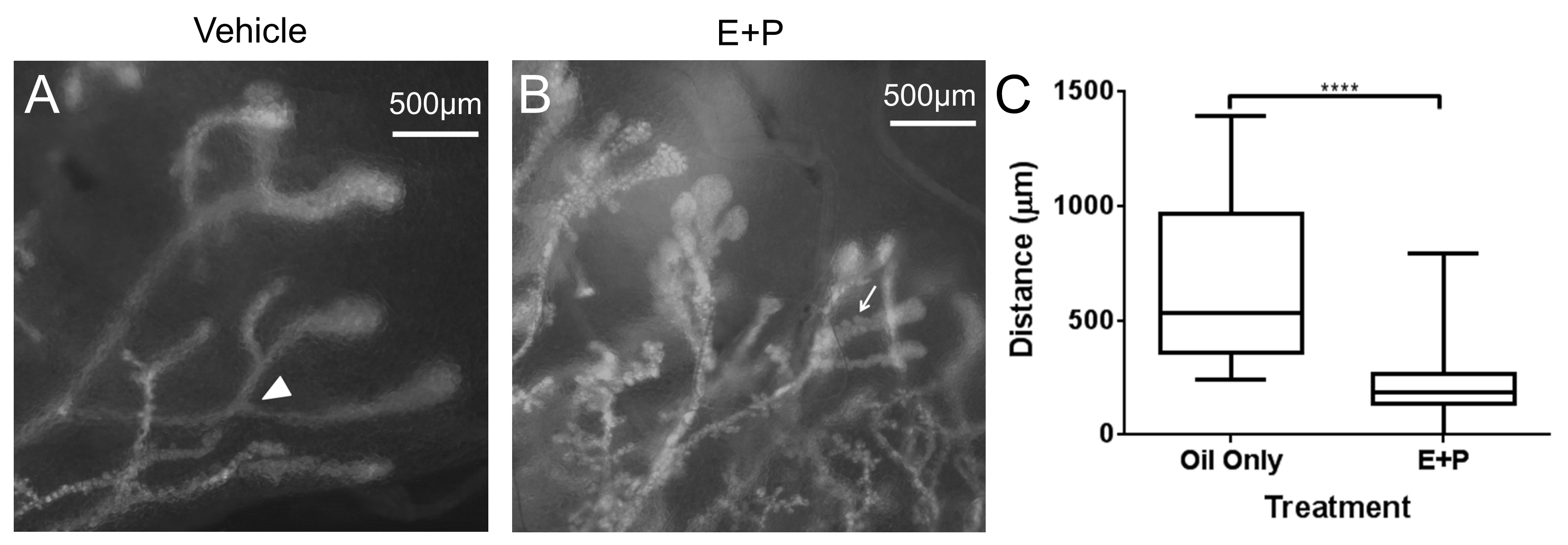

Supplement: S2 Fig — 5–6 week old mice were treated with estrogen and progesterone (E+P) in sesame oil or oil only for 10 days in order to induce alveologenesis in mature alveolar cells. The distances from the TEB to the first branchpoint or alveolar bud were measured. A) Representative image of a control treated gland with an arrow head indicating a branch point. B) Representative image of an E+P treated gland with an arrow indicating an alveolar bud. C) Quantification of the average distance between the TEB and branch/bud event as mean ±SEM; Control mean = 636.7μm ±59.28 n = 26, E+P mean = 216.48μm ±13.47 (p < .0001) n = 29. (TIF) [file pcbi.1004839.s002.tif]

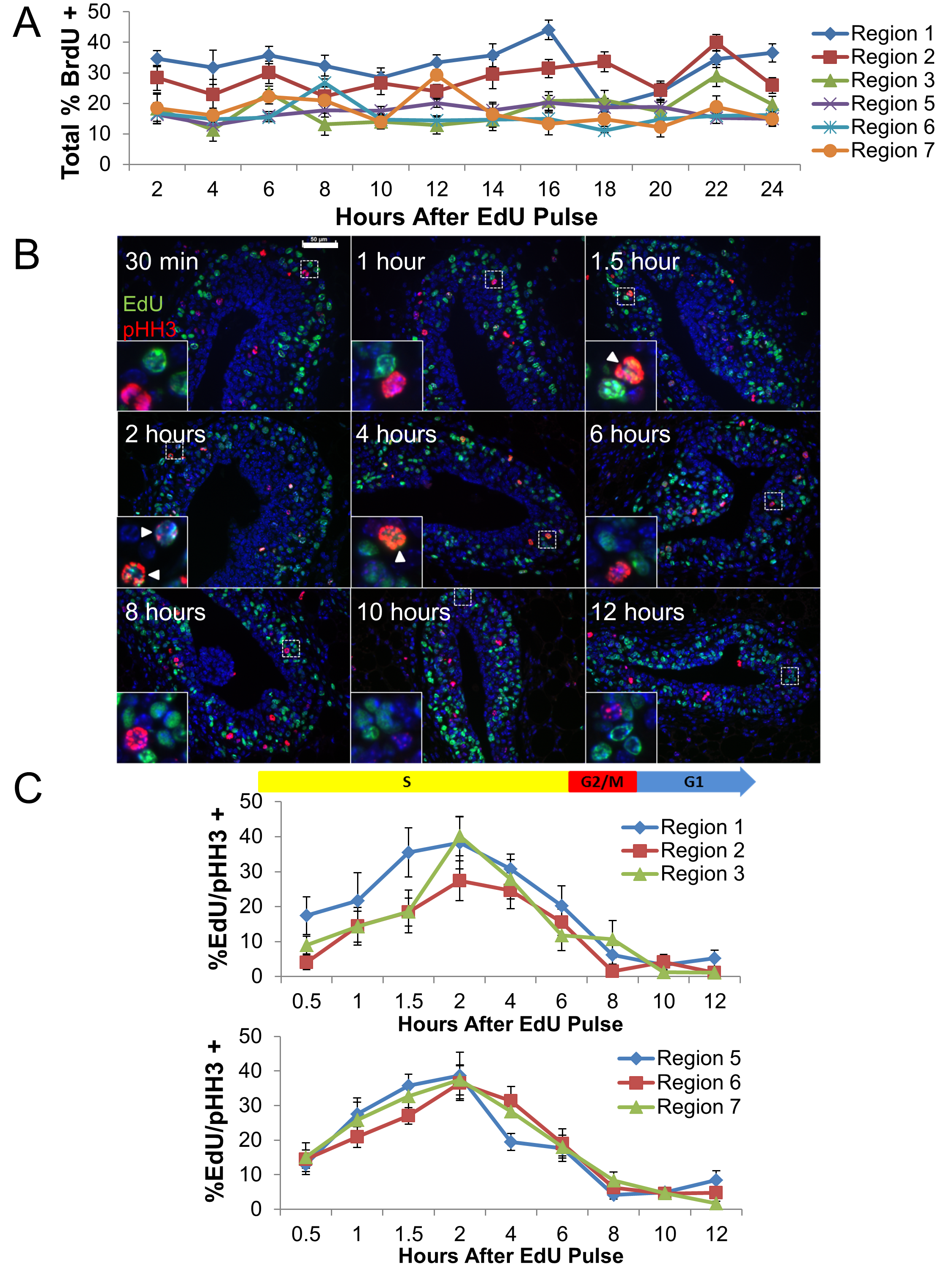

Supplement: S3 Fig — All mice were given a pulse of Edu at time 0 and BrdU two hours before harvest. Additional time points of 30 min, 1 hour and 1.5 hours was used for G2/M phase analysis. A) Total levels of BrdU are constant over time (each time point is mean ±SEM). B) Representative images of pHH3 and EdU co-staining. C) Quantification of EdU/pHH3 double positive cells by region over 12 hours indicate that duration of G2/M phase is 2 hours (each time point represents mean ±SEM, n = 10 TEBs per time point). (TIF) [file pcbi.1004839.s003.tif]

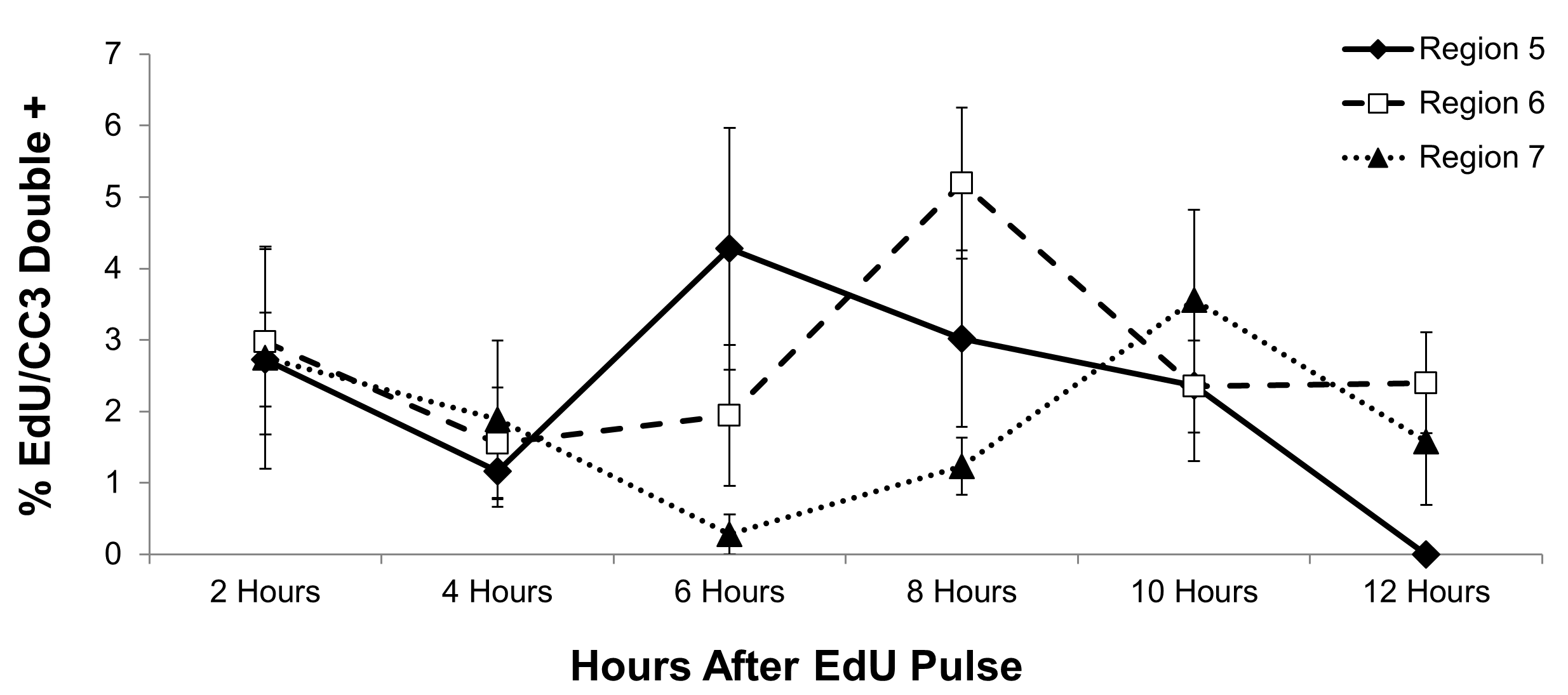

Supplement: S4 Fig — EdU pulsed cells were stained for CC3 to determine time to apoptosis. Quantification of EdU/CC3 double positive cells (mean ±SEM, n = 7–10 TEBs) indicates that most cells undergo apoptosis by 12 hours after EdU labelling. (TIF) [file pcbi.1004839.s004.tif]

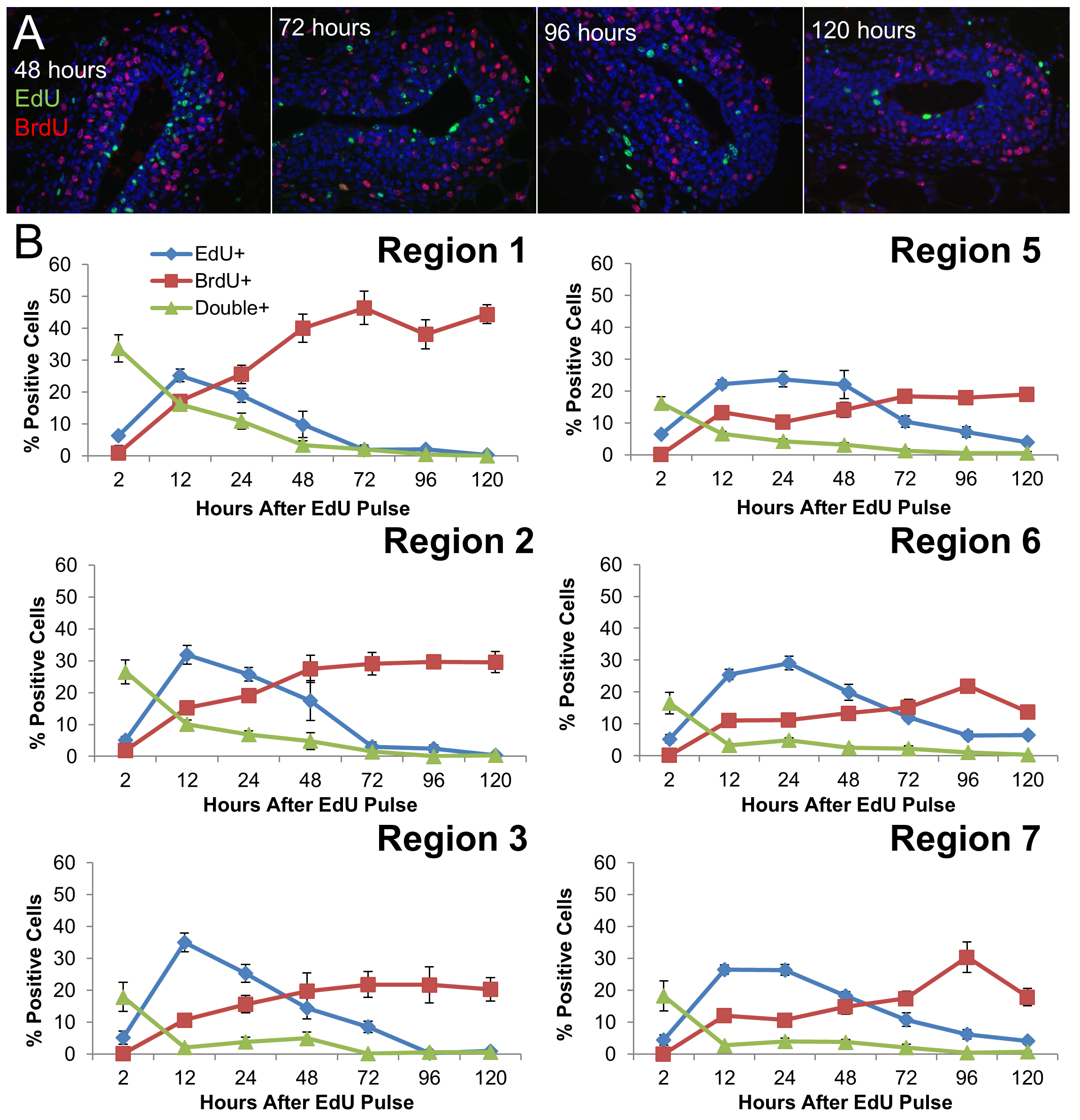

Supplement: S5 Fig — Mice were given a pulse of EdU at time 0 and glands were harvested every 24 hours (with a BrdU pulse given 2 hours before harvest) for up to 120 hours. A) Representative images of TEBs are shown including time points from previous pulse (2 and 12 hours). B) EdU and BrdU single and double positive cells were quantified by region up to 120 hours after EdU pulse (mean ±SEM, n = 7–10 TEBs). (TIF) [file pcbi.1004839.s005.tif]
