# Supplementary material for: A Geometrically-Constrained Mathematical Model of Mammary Gland Ductal Elongation Reveals Novel Cellular Dynamics within the Terminal End Bud
Source: PLoS Comput Biol. 2016 Apr 26;12(4):e1004839. doi: 10.1371/journal.pcbi.1004839 (PMC4845990; doi:10.1371/journal.pcbi.1004839)
Supplement: S1 Text — Tables of measurement values, experimental values, input parameter values, summary of predictions, and cell number by region validations are presented in tables A-E. Further explanations of mathematical derivations for A) Converting cell fractions into rates, B) Modeling cap cells dropping into the body cell layer, C) Correction factor for apoptosis rates in the body cell layer, D) Conversion from elongation to displacement rate, and E) General Formulation, parameters and validation are presented in the text. (DOCX) [file pcbi.1004839.s006.docx]

**Supplemental Information**

**Table A. Summary Values of All Experimental Measurements.** Measurements from Figures 1-4 are summarized as averages with SEM. ^a^ Numbers are sums of cell width and underlying region width. ^b^ Numbers are intra-nuclear distance.

|  | **Proliferation**  **(%BrdU+)** | **Cell Cycle Duration (hrs)** | **Apoptotic Index (AI)**  **(%CC3+)** | **Cell Width**  **(µm)** | **Cell Length**  **(µm)** | **Region Width**  **(µm)** | **Region Length**  **(µm)** | **Cell Number** |
| --- | --- | --- | --- | --- | --- | --- | --- | --- |
| Region 1 | 32.8 (±1.0) | 16 | 0.1 (±0.1) | 10.1 (±0.4) | 7.6 (±0.2) | 164.8^a^ | 85.8^a^ | 31.8 (±1.1) |
| Region 2 | 28.1 (±1.0) | 16 | 0.3 (±0.1) | 10.2 (±0.4) | 7.9 (±0.2) | 91.3/165.0^a^ | 156.9 (±13.6) | 37.3 (±1.2) |
| Region 3 | 17.5 (±1.0) | 16 | 0.5 (±0.3) | 9.6 (±0.4) | 10.0 (±0.2)^b^ | 90.1^a^ | 216.5 (±32.1) | 46.6 (±2.2) |
| Region 4 | NA | NA | - | 3.0 (±0.1) | 21.5 (±0.8)^b^ | 66.6^a^ | NA | NA |
| Region 5 | 17.4 (±0.5) | 16 | 4.0 (±0.5) | 11.1 (±0.2) | 7.1 (±0.1) | 144.7(±12.9) | 75.7 (±6.0) | 113.6 (±6.3) |
| Region 6 | 14.4 (±0.5) | 16 | 5.3 (±0.4) | 10.8 (±0.1) | 6.7 (±0.1) | 70.9/144.7 (±4.2/12.9) | 156.9 (±13.6) | 165.0 (±9.2) |
| Region 7 | 15.3 (±0.8) | 16 | 3.5 (±0.5) | 11.3 (±0.2) | 7.3 (±0.1) | 70.9 (±4.2) | 216.5 (±32.1) | 150.5 (±14.6) |
| Region 8 | NA | NA | - | 9.8 (±0.1) | 6.8 (±0.1) | 60.5 (±1.6) | NA | NA |

**Table B. Experimental Values Of Input Parameters For Initial Prediction Of Elongation Rates.** Apoptotic rates $d_{i}$ are evaluated as the ratio of the apoptotic index ${AI}_{i}$ experimentally measured in region $i$ and the apoptotic duration estimated as $\tau_{apoptosis}=12hrs\mp10\%$. Proliferation rates $r_{i}$ are evaluated in each region $i$ as the ratio of the proliferative index ${PI}_{i}$ derived from the fraction %$\mathrm{BrdU}_{i}^{+}$ of cells and the cell cycle duration $\tau_{cycle}=16hrs\mp10\%$.

|  |  | **Proliferation rate** (${day}^{-1}$) | | **Apoptotic rate** (${day}^{-1}$) | | **Cell number** ($\#$) | |
| --- | --- | --- | --- | --- | --- | --- | --- |
| **Region** | | $r_{i}$ | ${\Delta r}_{i}$ | $d_{i}$ | ${\Delta d}_{i}$ | $N_{i}$ | ${\Delta N}_{i}$ |
| Basal | 1 | 1.3124 | 0.1370 | 0.0018 | 0.0018 | 31.76 | 1.07 |
|  | 2 | 1.1224 | 0.1190 | 0.0065 | 0.0028 | 37.26 | 1.17 |
|  | 3 | 0.7000 | 0.0795 | 0.0094 | 0.0051 | 46.60 | 2.22 |
| Luminal | 5 | 0.6976 | 0.0730 | 0.0797 | 0.0132 | 113.56 | 6.29 |
|  | 6 | 0.5746 | 0.0609 | 0.1064 | 0.0137 | 164.95 | 9.24 |
|  | 7 | 0.6124 | 0.0695 | 0.0694 | 0.0115 | 150.50 | 14.56 |
|  |  |  |  |  |  | $\mathcal{l}$ | $\mathcal{\Delta l}$ |
| 4 | | **Myoepithelial cell length** $\mathcal{l}^{bas}$ ($\mu m$) | | | | 21.54 | 0.82 |
| 8 | | **Epithelial cell length** $\mathcal{l}^{lum}$ ($\mu m$) | | | | 6.84 | 0.07 |

**Table C. Experimental Values Of Additional Input Parameters For Modified Prediction Of Elongation Rates.** These values lead to a flux of cap cells dropping into the body cell layer $\varphi_{bas\to lum}=45.11\pm8.12 cells/day$ and an apoptotic correction factor $\delta=97\%$.

|  |  | **Apoptotic rate** (${day}^{-1}$) | | **Cap cell number** ($\#$) | | **Cap cell fraction** ($\%$) | |
| --- | --- | --- | --- | --- | --- | --- | --- |
| **Region** | | $d_{i}^{c}$ | ${\Delta d}_{i}^{c}$ | $N_{i}^{c}$ | ${\Delta N}_{i}^{c}$ | $\rho_{i}^{c}$ | ${\Delta\rho}_{i}^{c}$ |
| Luminal | 5 | 0.94 | 0.12 | 13.88 | 1.51 | 12.22 | 1.14 |
|  | 6 | 0.85 | 0.12 | 13.58 | 2.75 | 8.23 | 1.60 |
|  | 7 | 2.00 | 0.28 | 10.26 | 3.33 | 6.82 | 2.11 |

**Table D. Summary Of Predictions.** All rates are obtained using expressions (Eq.S9) and (Eq.S10). Elongation rates correspond to straight growth (*i.e.*, tortuosity $T=1$) and are converted into displacement rates using tortuosity $T=1.31\pm0.19$. Our initial prediction is obtained with a flux $\varphi_{bas\to lum}=0 cells/day$ and a correction factor $\delta=0\%$. Our modified prediction is obtained with a flux $\varphi_{bas\to lum}=45.11 cells/day$ and a correction factor $\delta=97\%$.

|  | **Elongation rate**  ($mm/day$) | | **Displacement rate**  ($mm/day$) | |
| --- | --- | --- | --- | --- |
| **Prediction** | Basal | Luminal | Basal | Luminal |
| Initial | $1.24\pm0.09$ | $0.78\pm0.07$ | $0.95\pm0.16$ | $0.60\pm0.10$ |
| Final | $0.76\pm0.12$ | $0.81\pm0.08$ | $0.58\pm0.13$ | $0.62\pm0.11$ |

**Table E. Comparison Of Theory To Experimental Cell Number In Each Region Of The TEB.** Theoretical cell numbers $N_{i}^{th}$ are obtained as the ratio of the region surface area to the average cell surface area and compared to directly measured cell numbers $N_{i}^{exp}$.

|  |  | **Theory** | | **Experiment** | | **Comparison** | |
| --- | --- | --- | --- | --- | --- | --- | --- |
| **Region** | | $N_{i}^{th}$ | $\Delta N_{i}^{th}$ | $N_{i}^{exp}$ | $\Delta N_{i}^{exp}$ | $({N_{i}^{th}-N}_{i}^{exp})/N_{i}^{exp}$ | |
| basal | 1 | 34.53 | 2.10 | 31.76 | 1.07 | 8.72 | % |
|  | 2 | 40.69 | 3.48 | 37.26 | 1.17 | 9.21 | % |
|  | 3 | 43.12 | 6.47 | 46.60 | 2.22 | 7.46 | % |
| luminal | 5 | 119.61 | 18.43 | 113.56 | 6.29 | 5.33 | % |
|  | 6 | 179.47 | 27.83 | 164.95 | 9.24 | 8.81 | % |
|  | 7 | 146.03 | 27.41 | 150.50 | 14.56 | 2.97 | % |

**Supplemental Text**

**(A) Conversion of stained cell fractions into rates**

By definition (see Macklin et al. [17] for example) the death (apoptotic) and birth (proliferation) rates, in each region $i$, are defined as the fractions of cells undergoing apoptosis ${AI}_{i}$(apoptotic index) and proliferative cells ${PI}_{i}$ (proliferative index) divided by the characteristic durations of apoptosis and cell cycle, respectively, *i.e.* :

$$d_{i}={AI}_{i}/\tau_{apoptosis}$$

and

$r_{i}={PI}_{i}/\tau_{cycle}$.

Apoptotic rates $d_{i}$are therefore directly evaluated from the experimental measurements (Fig 3, Table A) for the apoptotic duration experimentally estimated as $\tau_{apoptosis}=12hrs\mp10\%$ (S4 Fig).

Proliferation rates require the estimation of the proliferative index ${PI}_{i}$ (defined as the fraction of cycling cells, *i.e.* in phases G1, S, G2 and M). As BrdU incorporation only labels cells in S phase but not those which are in other phases yet still cycling, we used our measurements of the cell cycle phases and the fraction $\%\mathrm{BrdU}_{i}^{+}$of cells in S phase to find

${PI}_{i}=\frac{\tau_{cycle}}{\tau_{S phase}} \%\mathrm{BrdU}_{i}^{+}$ , and thus $r_{i}=\%\mathrm{BrdU}_{i}^{+}/\tau_{S phase}$

for the experimentally measured values of $\%\mathrm{BrdU}_{i}^{+}$ (Fig 2, Table A) and the S phase duration $\tau_{S phase}=6hrs\mp10\%$ (Fig 2).

**(B) Modeling cap cells dropping into the body cell layer**

We account for cap cells dropping into the body cell layer by starting with the balanced equation (Eq.1) for cell number $N_{i}$ in region $i$, which we recall below:

| $\frac{dN_{i}}{dt}=0=+\sum_{l} \varphi_{l\to i}-\sum_{l} \varphi_{i\to l}+r_{i}N_{i}-d_{i}N_{i}$ | (Eq.S1) |
| --- | --- |

where (see S1 Fig):

- As originally, non-zero fluxes to be considered are $\varphi_{1\to2}$, $\varphi_{2\to3}$ and $\varphi_{3\to4}$ within the basal layer, and $\varphi_{5\to6}$, $\varphi_{6\to7}$ and $\varphi_{7\to8}$ within the luminal layer;
- additional non-zero fluxes $\varphi_{1\to5}$, $\varphi_{2\to6}$ and $\varphi_{3\to7}$ model cap cells movement from the basal to the luminal layer of the TEB, which sum to $\varphi_{bas\to lum}=\varphi_{1\to5}+\varphi_{2\to6}+\varphi_{3\to7}$.

Following the original derivation, the modified cell fluxes from the TEB to the mature duct read:

- for the elongation of the basal layer

| $\varphi_{3\to4}=\sum_{i=1,2,3} \left( r_{i}-d_{i} \right)N_{i}-\left( \varphi_{1\to5}+\varphi_{2\to6}+\varphi_{3\to7} \right)= \Phi^{bas}-\varphi_{bas\to lum}$ | (Eq.S2) |
| --- | --- |

- for the elongation of the luminal layer

| $\varphi_{7\to8}=\sum_{i=5,6,7} (r_{i}-d_{i})N_{i}+\left( \varphi_{1\to5}+\varphi_{2\to6}+\varphi_{3\to7} \right)= \Phi^{lum}+\varphi_{bas\to lum}$ | (Eq.S3) |
| --- | --- |

We propose to derive a mathematical expression of $\varphi_{bas\to lum}$ based on the biological observations that cap cells dropping into the body cell layer are no longer proliferative and are actively undergoing apoptosis. Such a derivation requires the description of two cell types within the body cell layer. Thus, in the following, we consider values of the subscript $i\in\left\{ 5,6,7 \right\}$, which define the regions of the body cell layer, and we introduce:

- $N_{i}^{c}$: the number of “cap cells within the body cell layer”, *i.e.*, cells originated from the cap cell layer and found in the body cell layer;
- $N_{i}^{b}$: the number of “true body cells”, *i.e.*, cells originally from and remaining in the body cell layer.

Similar to Eq.S1, a balance of cell number of each type can be performed in every region of the body cell layer; Thus, for $i\in\left\{ 5,6,7 \right\}$:

| $\left\{ \begin{matrix} \frac{dN_{i}^{b}}{dt}=0=+ \varphi_{i-1\to i}^{b}-\varphi_{i\to i+1}^{b}+\left( r_{i}^{b}-d_{i}^{b} \right)N_{i}^{b} \\ \frac{dN_{i}^{c}}{dt}=0=+ \varphi_{i-1\to i}^{c}-\varphi_{i\to i+1}^{c}+ \varphi_{i-4\to i}+\left( r_{i}^{c}-d_{i}^{c} \right)N_{i}^{c} \end{matrix} \right.$ | (Eq.S4) |
| --- | --- |

with the convention that $\varphi_{4\to5}^{b}=\varphi_{4\to5}^{c}=0$ and $\varphi_{i-4\to i}, i\in\left\{ 5,6,7 \right\}$, the influxes from the cap cell layer. This system can actually be rewritten as Eq.S1 when gathering the two cell types into a single cell population, hence considering $N_{i}=N_{i}^{b}+N_{i}^{c}$ and ${\varphi_{i\to j}=\varphi}_{i\to j}^{b}+\varphi_{i\to j}^{c}$, then defining the cap cell fraction in each region $i$ as $\rho_{i}^{c}=N_{i}^{c}/N_{i}$, and finally using the classical mixture representation to define the average population proliferation and apoptotic rates as:

| $r_{i}=\left( 1-\rho_{i}^{c} \right)r_{i}^{b}+\rho_{i}^{c} r_{i}^{c}$ and $d_{i}=\left( 1-\rho_{i}^{c} \right)d_{i}^{b}+\rho_{i}^{c} d_{i}^{c}$ | (Eq.S5) |
| --- | --- |

We use the second line of Eq.S4 together with the following experimentally observed features of cap cells, which we assume in our derivation:

- cap cells within the body cell layer do not proliferate (*i.e.*, $r_{i}^{c}=0, i\in\left\{ 5,6,7 \right\}$);
- cap cells within the body cell layer are absent from the luminal layer of the mature duct, which leads to $\varphi_{7\to8}^{c}=0$;

This leads to the expression of the total flux of cap cells from the basal to the luminal layer:

| $\varphi_{bas\to lum}= \sum_{i=5,6,7} {d_{i}^{c}N}_{i}^{c}$ | (Eq.S6) |
| --- | --- |

This flux corresponds to all cap cells found in the body cell layer not contributing to the elongation of the duct but dying within the luminal regions of the TEB.

**(C) Correction factor for apoptosis rates in the body cell layer**

In each region $i\in\left\{ 5,6,7 \right\}$, for known values of the cap cell fraction $\rho_{i}^{c}$ and the average apoptotic rates $d_{i}$ and $d_{i}^{c}$ for the total population and cap cell population only, respectively, one can rewrite the right hand part of Eq.S5 to obtain the true body cell apoptotic rate:

| $d_{i}^{b}=\frac{d_{i}-\rho_{i}^{c} d_{i}^{c}}{1-\rho_{i}^{c}}$ | (Eq.S7) |
| --- | --- |

When checking for model consistency, we realized that this expression could become negative when evaluated using our experimental measurements (See Table C). This could be the result of overestimating the cap cell fraction, or the average cap cell apoptotic rate. However, as our staining for cell death corresponds to mid stage apoptosis only, and is about 50% the rate of TUNEL staining by Humphreys et al. (late stage apoptosis), we suggest that the average experimentally measured apoptotic rate $d_{i}$ is actually underestimated and we introduce the modified apoptotic rate $\hat{d_{i}}=\left( 1+\delta_{i} \right)d_{i}$ with correction factor $\delta_{i}$. The simplest criterion to evaluate this factor is by imposing the positivity of the right hand side of Eq.S7, hence providing a minimal value $\delta_{i}$ in each luminal region of the TEB. For simplicity, we consider a uniform correction factor $\delta$ over the three luminal regions, which we define as

| $\delta=\max_{i=5,6,7} \left( \rho_{i}^{c}\frac{d_{i}^{c}}{d_{i}}-1 \right)$ | (Eq.S8) |
| --- | --- |

which ensures all positive values for each individual region.

**(D) Conversion from elongation to displacement rate**

Tortuosity refers to the characteristic of a curved path as opposed to a straight one. It is defined as the arc:chord ratio $T=L/D$ where $L$ is the arc length (*i.e.* the length of the real path) and $D$ is the chord length (*i.e.* the displacement given by the start-to-end straight line) (Fig. 6C).

As the elongation rate $\lambda_{elong}$ in our mathematical model corresponds to a time-independent displacement rate in a specific direction, it can be written as $\lambda_{elong}=L/\Delta t$ for a path of length $L$ covered over a period of time $\Delta t$, while the corresponding displacement rate, experimentally measured, is $\lambda_{disp}=D/\Delta t$.

Therefore tortuosity is also the elongation:displacement ratio, $T=\lambda_{elong}/\lambda_{disp}$, which provides us with the method required for converting the model value ${(\lambda}_{elong})$ to the measurement value ${(\lambda}_{disp})$.

Total tortuosity of the trajectory of a single TEB within the ductal tree comes from two observations. First, the direction of duct elongation is modified at each bifurcation point (Fig. 6A), making the TEB trajectory oscillating around a mean direction with deflection angle $\alpha$ measured experimentally ($\alpha=35.5^{\circ}$) (Fig. 6B). For simplicity, we assume that such oscillations can be represented using a cosine function, which leads to an mean tortuosity of $T_{bifurcation}=1/\cos\left( \alpha\right)(=1.23)$.

Second, the duct grows in a locally curved manner (Fig 6C), which leads to a local tortuosity of $T_{curvature}=1/(1-\Delta)(=1.06)$ between two consecutive bifurcation points, $\Delta$ being measured experimentally ($\Delta=6.13\%$) (Fig 6E). The combination of both effects results in total tortuosity $T=1/(\left( 1-\Delta\right)\cos\left( \alpha\right)) (=1.31)$ which we use to convert the mathematically predicted elongation rate $\lambda_{elong}$ into the experimentally measured displacement rate $\lambda_{disp}$ for model validation.

**(E) Mathematical modeling of ductal elongation: general formulation, parameters and validation**

**1) General formulation.** For clarity and reader’s convenience we start with gathering the various parts of our mathematical derivation into the most complete and general formulation of the basal and luminal displacement rates, hence summarizing our model as:

| $\lambda^{bas}=\frac{\mathcal{l}^{bas}}{2T}\left( \sum_{i=1,2,3} \left( r_{i}-d_{i} \right)N_{i}-\varphi_{bas\to lum} \right)$ | (Eq.S9) |
| --- | --- |

| $\lambda^{lum}=\frac{\mathcal{l}^{lum}}{2T}\left( \sum_{i=5,6,7} \left( r_{i}-d_{i}\left( 1+\delta\right) \right)N_{i}+\varphi_{bas\to lum} \right)$ | (Eq.S10) |
| --- | --- |

where the required input can be separated into

*i) parameters for initial prediction – Model 1 (see Table B & S1E2a Text) :*

- $\mathcal{l}^{bas}$ and $\mathcal{l}^{lum}$ : myoepithelial (basal) and epithelial (luminal) cell lengths, respectively ;
- $r_{i}$ and $d_{i}$ : average population growth (proliferation) and death (apoptotic) rates, respectively, in Region $i$;
- $N_{i}$ : cell number in Region $i$.

*ii) parameters for modified predictions (S1E2b Text) :*

- $\varphi_{bas\to lum}$ : flux of cap cells dropping into the body cell layer (Model 2);
- $\delta$ : correction factor for apoptotic index in the luminal regions (Model 3).

*iii) conversion elongation-displacement rate – Model 4 (S1D Text) :*

- $T$ : average tortuosity of a single duct.

**2) Model parameters and corresponding predictions**

*a) Initial prediction*

Our first prediction does not account for tortuosity of the duct and therefore refers to elongation rates (*i.e.,* total new duct length). Additionally, this prediction does not account for cap cells dropping into the body cell layer (*i.e.*, $\varphi_{bas\to lum}=0 cells/day$) and the requirement of a correction factor for apoptotic index in the luminal regions is consequently irrelevant (*i.e.*, we consider $\delta=0\%$). All parameters needed at this step are presented in Table B. The corresponding elongation and displacement rates are summarized in Table D.

*b) Final prediction accounting for* $\varphi_{bas\to lum}$ *and its consequences*

We maintain the parameter values required for our initial prediction. The modified prediction requires knowing the values of two additional parameters $\varphi_{bas\to lum}$ and $\delta$ that can be estimated using Eq.S6 and Eq.S8, respectively. Using parameters presented in Table C, we find $\varphi_{bas\to lum}=45.11\pm8.12 cells/day$ and $\delta=97\%$. The corresponding elongation and displacement rates are summarized in Table D.

The flux $\varphi_{bas\to lum}$ is the number of newborn cap cells dropping indifferently from the basal regions $i\in\left\{ 1,2,3 \right\}$ into the luminal regions $i\in\left\{ 5,6,7 \right\}$ by unit time. It is interesting to study the proportion of newborn cap cells actually dropping into the luminal layer (see S1 Fig). Indeed, if we assume that cap cells drop only from Region 1 into Region 5 (*i.e.* $\varphi_{2\to6}=\varphi_{3\to7}=0$), this proportion would be defined as the ratio $\varphi_{bas\to lum}/r_{1}N_{1}$ whose value is larger than one which is physically impossible. This remark has important biological implications as it shows that cap cells must also drop from Region 2 into Region 6 (*i.e.*, $\varphi_{2\to6}\neq0$). We then define the proportion of newborn cap cells in Regions 1 and 2 dropping into Regions 5 and 6 as

| $X^{*}=\frac{\varphi_{bas\to lum}}{r_{1}N_{1}+r_{2}N_{2}}=54.02\pm10.59$ | (Eq.S11) |
| --- | --- |

Such a proportion could also be based on considering $\varphi_{3\to7}\neq0$ and would thus be defined as $\varphi_{bas\to lum}/\left( r_{1}N_{1}+r_{2}N_{2}+r_{3}N_{3} \right)$. We would find the value $38.85\pm7.44$, which would not bring any additional insight.

**3) Model validations**

*a) Checking the time-invariant constrained TEB geometry*

Our model is based on considering the stationary morphology of the TEB shown in Fig. 2. In order to validate our approach, we evaluate the number of cells in each of the six TEB regions in a 2D cross-section, *i.e.*, $i\in\left\{ 1,2,3,5,6,7 \right\}$, as the ratio of the region surface area $A_{i}$ by the single cell characteristic surface area $S_{i}$. It is straightforward to evaluate $A_{i}$ for each region due to the simplicity of the geometrical surfaces (see main text), whereas we consider cells as deformable ellipses with axis dimensions given by the cell length and width measured experimentally in each region. Comparison of the theoretical cell numbers $N_{i}^{th}=A_{i}/S_{i}$ to the experimental cell numbers $N_{i}^{exp}$ is presented in Table E and shows an excellent agreement.

*b) Fit versus data-informed results*

We have shown in the previous supplemental sections that our predictions are entirely informed by our experimental data. In particular, we have found numerical values for the flux $\varphi_{bas\to lum}$ and the apoptotic correction factor$\delta$ using mathematical modeling informed by our experimental data. In order to validate our approach, we show that values of $\varphi_{bas\to lum}$ and $\delta$ can also be estimated by using a fitting process, which will lead to very similar results to the ones we obtain with a fully data-driven model.

As stated in the main text, the basal and luminal elongation rates must match since the two layers are adhered to one another. Therefore, we consider here $\varphi_{bas\to lum}$ and $\delta$ as free parameters. For a fixed value of $\delta$, $\varphi_{bas\to lum}$ can be uniquely determined using the matching condition $\lambda^{bas}=\lambda^{lum}$ where both rates are given by Eq.S9-S10. Results presented in Figure 7 illustrate that such a fit provides values of the elongation rates that lay within the error range of our modeling approach, thus strengthening its validity.
